# Unscheduled DNA synthesis leads to elevated uracil residues at highly transcribed genomic loci in *Saccharomyces cerevisiae*

**DOI:** 10.1371/journal.pgen.1007516

**Published:** 2018-07-17

**Authors:** Norah Owiti, Shanqiao Wei, Ashok S. Bhagwat, Nayun Kim

**Affiliations:** 1 Department of Microbiology and Molecular Genetics, University of Texas Health Science Center at Houston, Houston, TX United States of America; 2 The University of Texas Graduate School of Biomedical Sciences, Houston, TX United States of America; 3 Department of Chemistry, Wayne State University, Detroit, MI United States of America; 4 Department of Biochemistry, Immunology and Microbiology, Wayne State University, Detroit, MI United States of America; National Institute of Environmental Health Sciences, UNITED STATES

## Abstract

Recombination and mutagenesis are elevated by active transcription. The correlation between transcription and genome instability is largely explained by the topological and structural changes in DNA and the associated physical obstacles generated by the transcription machinery. However, such explanation does not directly account for the unique types of mutations originating from the non-canonical residues, uracil or ribonucleotide, which are also elevated at highly transcribed regions. Based on the previous findings that abasic (AP) lesions derived from the uracil residues incorporated into DNA in place of thymine constitute a major component of the transcription-associated mutations in yeast, we formed the hypothesis that DNA synthesis ensuing from the repair of the transcription-induced DNA damage provide the opportunity for uracil-incorporation. In support of this hypothesis, we show here the positive correlation between the level of transcription and the density of uracil residues in the yeast genome indirectly through the mutations generated by the glycosylase that excise undamaged cytosine as well as uracil. The higher uracil-density at actively transcribed regions is confirmed by the long-amplicon PCR analysis. We also show that the uracil-associated mutations at a highly transcribed region are elevated by the induced DNA damage and reduced by the overexpression of a dUTP-catalyzing enzyme Dut1 in G1- or G2-phases of the cell cycle. Overall, our results show that the DNA composition can be modified to include higher uracil-content through the non-replicative, repair-associated DNA synthesis.

## Author summary

Uracil in DNA, a major source of spontaneous mutations, can occur through the deamination of cytosine residues or through the direct incorporation of dUTP by DNA polymerases. Recent studies in yeast have shown that the uracil-associated mutations occur more frequently at highly transcribed regions. Because the reduction in dUTP pool decreased these mutations, it was postulated that the extent of uracil-incorporation into DNA is significantly affected by the local transcription activity. We show here that the higher transcription rate does correlate with the higher uracil-density in the yeast genome. We further provide multiple lines of evidence supporting a model of uracil-incorporation into DNA that is dependent on the repair synthesis of transcription-associated DNA damage.

## Introduction

Transcription, a fundamental cellular process, can incongruously pose a serious threat to genome stability. Highly transcribed genomic regions have been reported to be hotspots for mutagenesis and recombination, phenomena referred to as transcription-associated mutagenesis (TAM) and transcription-associated recombination (TAR), respectively [1–4]. Several different ways by which transcription promotes genomic instability have been described. First, the single strand DNA generated by DNA strand-separation during transcription is much more chemically labile than double-stranded DNA, leading to the mutations resulting from the spontaneous base modifications such as deamination [5–8]. Second, transcription necessitates a change in DNA topology and the ensuing accumulation of both negative and positive supercoils promotes the formation of non-canonical secondary structures such as R-loops, the stable hybrids of transcribed DNA and nascent RNA or G-quadruplex DNAs (G4 DNA), the four-stranded DNA configuration held together by Hoogsteen bonds among guanine bases [9, 10]. These structures leave the non-transcribed DNA in the susceptible single-stranded state and frequently lead to stalling and eventual collapse of the replication fork, which must be restarted/ repaired *via* homologous recombination [11–13]. Finally, because replication and transcription occur on the same template, collisions between the replication fork and transcription machinery are possible [14, 15]. These collisions induce helical stress and can trigger replication fork reversal giving rise to non-canonical DNA structures that can be processed into double stranded breaks [16, 17].

Recently, a novel mechanism of transcription-associated mutagenesis involving the non-canonical DNA nucleotide, uracil, was identified through genetic studies in yeast [18, 19]. Due to its close structural resemblance to thymine, uracil can be directly incorporated into DNA by DNA polymerases that cannot distinguish between the two bases, leading to U:A base mispairs [20]. Subsequent uracil removal by an uracil-DNA glycosylase (Ung1 in yeast) creates potentially toxic apurinic/apyrimidinic (AP) sites [21]. AP sites are the most prevalent endogenous DNA lesions produced from either spontaneous or DNA N-glycosylases-catalyzed hydrolysis of the base-glycosidic linkage and can act as a potent block to the transcription machinery and the replicative DNA polymerases. [22]. Blocked DNA synthesis can be rescued by the recruitment of translesion synthesis (TLS) DNA polymerases Rev1 and Polζ that together bypass the lesion by typically incorporating a C nucleotide across from the AP site in yeast and metazoans [23–26]. In addition to their misincorporation into DNA by DNA polymerases during replication or repair, uracil in DNA can result from either spontaneous or enzymatic deamination of cytosines to create U:G mispairs, which cause G:C to A:T transitions. Although uracil in DNA leads to deleterious mutations if not properly repaired, at the antibody-encoding immunoglobulin gene, it is an essential intermediate in antibody affinity maturation during the adaptive diversification processes [27].

The detrimental mutagenic outcome of uracil in DNA is prevented by Base Excision Repair (BER) pathway, which initiates repair *via* the AP endonuclease-catalyzed cleavage of the sugar-phosphate backbone at the 5’ side of the AP lesion. In yeast, the major AP endonucleases are Apn1, which carries out 95% of the repair, and Apn2 [28, 29]. N-glycosylases such as Ntg1 and Ntg2 with the associated AP lyase activity can also create breaks at the DNA backbone adjacent to the AP site in certain instances such as when the AP endonucleases activity is diminished or overwhelmed [19, 30]. Subsequent steps in BER involve the removal of the blocked DNA ends, gap filling by a DNA polymerase and ligation of the remaining nick by a DNA ligase. Although Nucleotide Excision Repair (NER) usually removes bulky, helix-distorting lesions such as UV-induced DNA damage, the transcription-coupled repair (TCR) sub-pathway of NER has been implicated in the repair of AP sites when BER is overburdened or disrupted [19]. TCR specifically repairs the RNA polymerase-stalling lesions in the transcribed strand of active genes encouraging a rapid removal of damage and preventing the accumulation of mutations on the transcribed strand. In yeast, Rad14 is absolutely required for TCR repair of AP sites, while Rad26 and Def1 contribute partially [19, 31].

Deoxyuridine triphosphatase (dUTPase), a ubiquitous enzyme that is essential for viability in both prokaryotic and eukaryotic organisms, catalyzes the conversion of dUTP to dUMP and pyrophosphate (Ppi), thereby reducing the pool of free dUTP and preventing the incorporation of uracil into DNA [32]. Following the conversion of dUTP to dUMP by the dUTPase, Thymidylate Synthase (TS), using tetrahydrofolate as a methyl donor, converts dUMP to dTMP, an intermediate that is required for dTTP synthesis. dUTPase serves two essential functions; maintaining a low intracellular [dUTP]/[dTTP] ratio, thus minimizing incorporation of uracil into DNA, and providing an important intermediate, dUMP, for the *de novo* synthesis of thymidylate. In addition to pyrophosphorlysis of dUTP by dUTPase, the pools of dUMP required for TS reaction is partly supplied by the deamination of dCMP by deoxycytidylate deaminase, Dcd1 in yeast [33].

Recent studies in *Saccharomyces cerevisiae* demonstrated an increase in uracil-derived mutations following the activation of transcription at a defined reporter gene [18]. These mutations were highly elevated by the disruption of BER and eliminated by the deletion of *UNG1* gene or the overexpression of yeast dUTPase, Dut1, suggesting that the uracil-dependent mutations result from the AP sites generated by excision of uracil incorporated into DNA. Repressing the transcription of the reporter gene lowered the uracil-associated mutations, suggesting a link between the extent of uracil incorporation into DNA and the process of transcription. However, a clear demonstration of higher uracil content at highly transcribed genomic loci is still lacking. In addition, how the nucleotide composition is affected by transcription remains to be deciphered. In the current study, we used the mutagenesis reporter with a regulatable promoter to further investigate the link between active transcription and uracil DNA content. Our results show that there are significantly more uracil residues present at the highly transcribed genomic locus and that the DNA glycosylase activity is slightly enhanced when transcription is elevated. Furthermore, we show that the DNA repair synthesis, induced by DNA damaging agents, leads to an increase in uracil residues present in DNA and that the overexpression of Dut1 in G1- and G2-phases of the cell cycle leads to a significant reduction in the uracil-dependent mutations at the highly transcribed site. Overall, our data strongly support a model where various transcription-associated damage induce unscheduled DNA synthesis, particularly in G1 and G2, subsequently leading to the elevated uracil residues at highly transcribed genomic loci.

## Results

**Fig 1:**
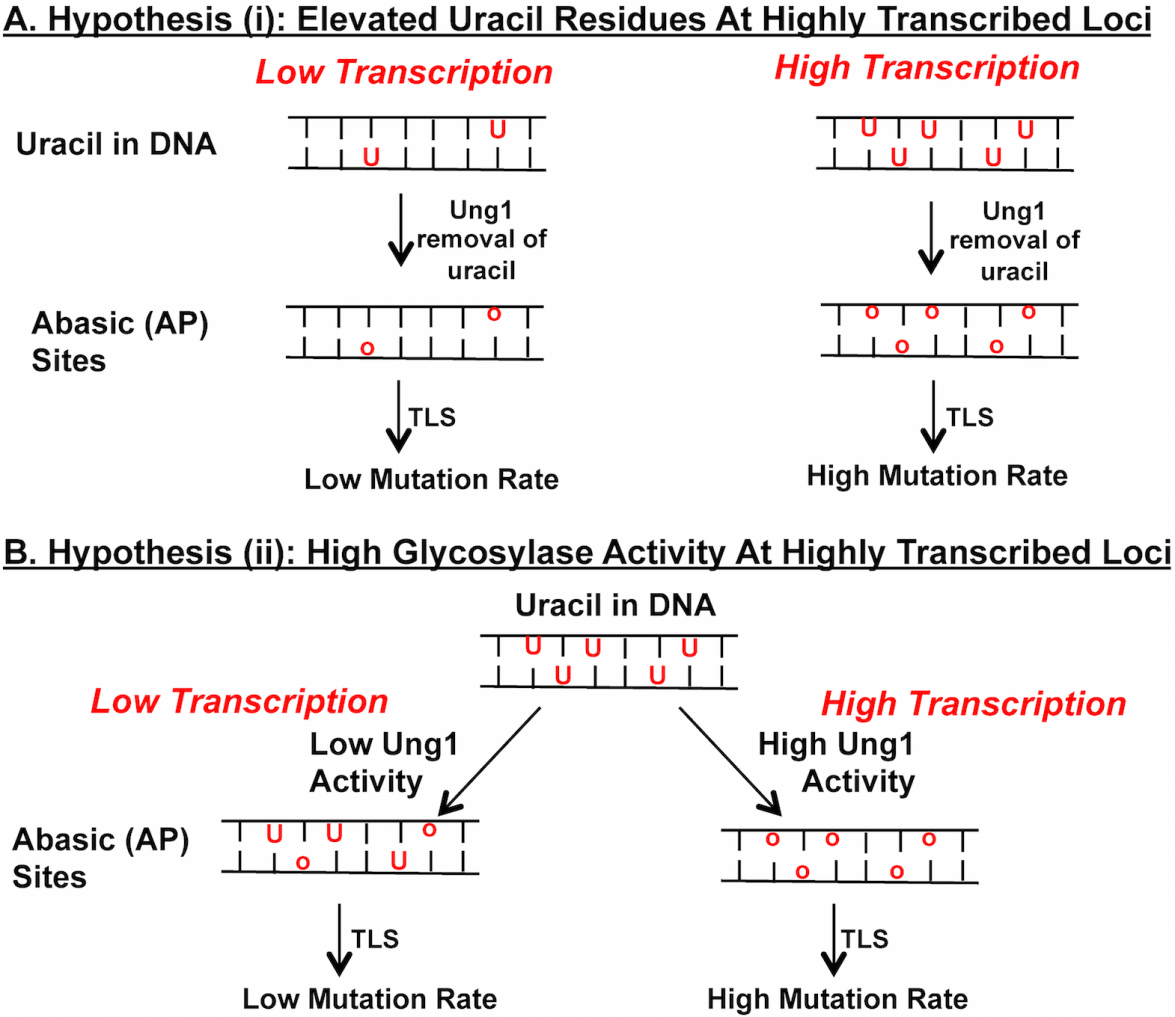
Alternative hypotheses for the transcription-associated elevation in uracil-dependent mutations. **A)** Higher number of uracil residues are present at highly transcribed loci resulting in the higher rate of uracil-dependent mutations. **B)** Uniform number of uracil residues are present regardless of the transcription level; the uracil DNA glycosylase activity is enhanced at highly transcribed genomic loci. In both instances, the uracil residue (***U***) is recognized and excised by Ung1 to create AP sites (***o*)**. The resulting abasic site is bypassed by translesion synthesis (***TLS***) polymerases in a mutagenic manner.

Previously, a mutation reporter system with a tetracycline-regulatable promoter *(pTET)* was used to examine the effect of transcription on the mutagenesis in BER/NER-deficient yeast strains [19]. Specifically, in the *pTET-lys2-TAA* system, the in-frame insertion of “TAA” stop codon disrupting the *LYS2* ORF renders the yeast cells auxotrophic for lysine (Lys^-^). Those mutations abrogating the TAA stop codon and allowing the translation read-through are then selected by the reversion to Lys+ phenotype. In the repair-deficient, *apn1Δ*, *apn1Δ rad14Δ*, and *apn1Δ ntg1Δ ntg2Δ* backgrounds, we observed a dramatic elevation in mutagenesis, particularly of A>C and T>G transversions, when the *pTET* promoter was activated. These mutations were mostly eliminated when Ung1 was disabled, or when transcription was repressed by the addition of the tetracycline analog doxycycline (+DOX). The rate of A>C and T>G mutations was also significantly reduced when the dUTPase-encoding *DUT1* gene was highly overexpressed to reduce the level of free dUTP available for incorporation into the genome. When uracil is misincorporated in place of thymine in DNA and subsequently excised by Ung1 to generate AP sites, the net result of Rev1/Polζ-dependent translesion bypass synthesis, inserting predominantly C nucleotides opposite AP sites, is expected to be A>C or T>G mutations. Therefore, the highly elevated rate of A>C and T>G mutations we observed under high transcription conditions at the *pTET-lys2-TAA* reporter is originating from the uracil residues in DNA that are excised by Ung1 to generate the mutagenic AP sites. There are two possible explanations for the enhanced uracil-dependent mutations when transcription is activated: (i) highly active transcription leads to elevated dUTP incorporation into the genome or, (ii) rather than affecting the number of uracil residues incorporated into the DNA, active transcription leads to the enhanced uracil glycosylase activity. These two hypotheses, each leading to the accumulation of mutagenic AP sites specifically at highly transcribed genes, are summarized in Fig 1.

### CDG glycosylase causes uracil- and cytosine- derived mutations

**Fig 2:**
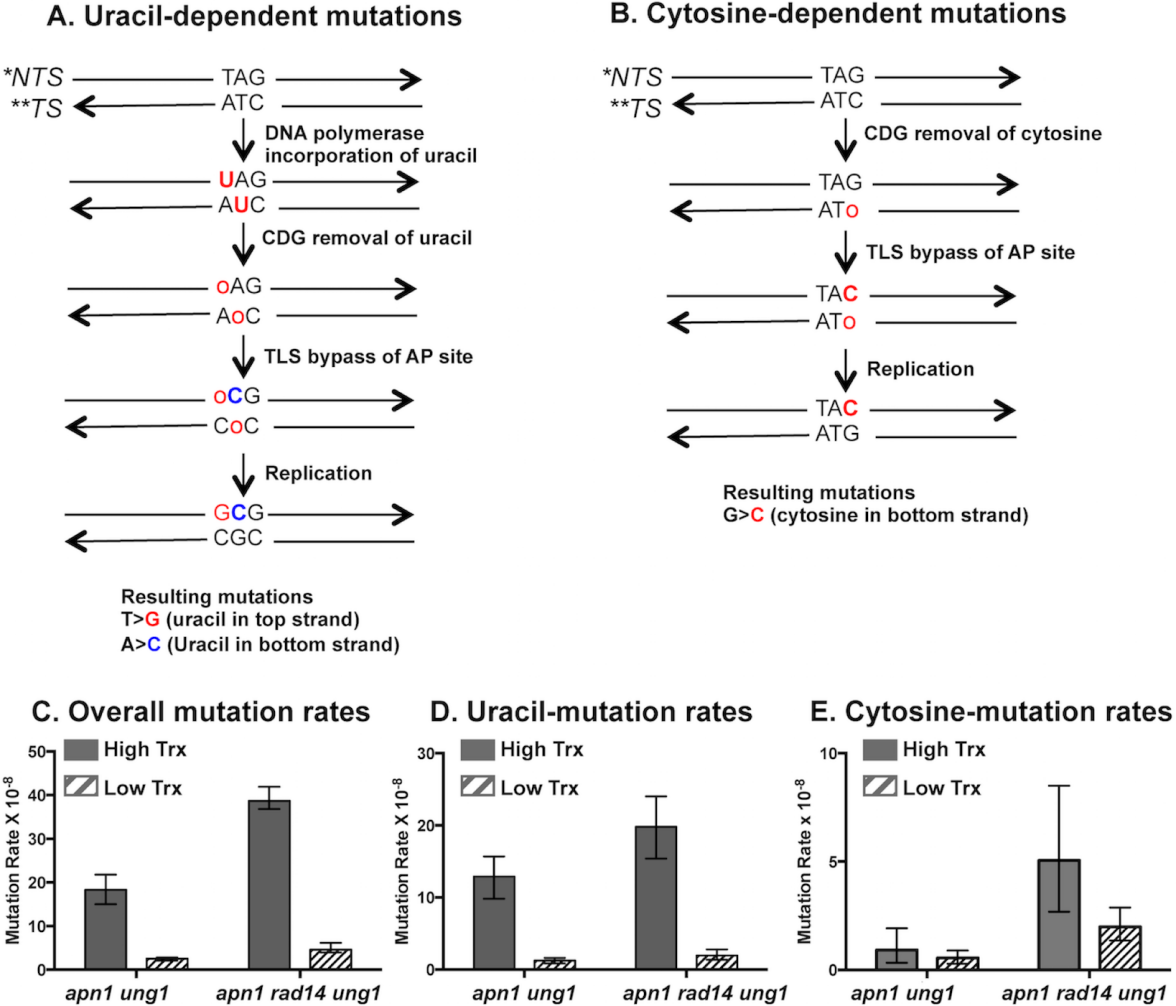
The effect of CDG expression on uracil- and cytosine-derived mutations. Schematic representations of the mutations originating from **A)** the uracil and **B)** the cytosine excised by the CDG glycosylase. Uracil or cytosine residue is removed by CDG creating an abasic site. The AP site (***o***) is bypassed by TLS polymerases inserting predominantly C across the AP site. *NTS; non-transcribed strand, **TS; transcribed strand. **C)** Overall mutation rates in yeast strains with CDG glycosylase-expressing plasmid under high-transcription (no doxycycline) or low-transcription (doxycyline added) conditions. Error bars represents 95% confidence intervals. **D)** and **E)** Rates of the uracil-dependent A>C and T>G mutations and the cytosine-dependent G>C mutations, respectively. See MATERIALS AND METHODS for the calculation of these rates. The numerical values of the median mutation frequencies and the confidence intervals represented as graphs in this figure are listed in S2 Table. All mutation rates are calculated from 24 independent cultures.

In order to investigate whether the main cause underlying the transcription-associated elevation in uracil-dependent mutations is the higher uracil density or the enhanced uracil excision, we used a modified human uracil DNA glycosylase (UDG). The mutant enzyme, hereon referred to as CDG, was generated by introducing Asn204 to Asp mutation in the substrate binding pocket of the UDG [34, 35]. CDG is able to excise unmodified cytosine residues from oligonucleotide substrates *in vitro*. Expression of CDG in the TLS-proficient yeast cells was previously shown to induce the accumulation of A:T > C:G and G:C > C:G transversions, resulting from the excision of uracils and cytosines, respectively. We expressed CDG to generate AP sites through excision of cytosines and uracils in yeast cells containing the mutation reporter *pTET-lys2-TAG*. This reporter contains the in-frame TAG stop codon inserted into the *LYS2* ORF and mutations at this stop codon is required for the reversion to Lys^+^ phenotype. The *pTET-lys2-TAG* reporter can be transcribed at a high or low level by the absence or presence of doxycycline in the media, respectively. Like the *pTET-lys2TAA* reporter used in previous studies of uracil-associated mutations under high transcription conditions, the *pTET-lys2-TAG* reporter is helpful in identifying uracil-associated mutations and in addition allow the detection of mutations arising from the excision of cytosines. As illustrated in Fig 2A, a A>C or T>G mutation is expected when an AP site is generated from the excision of uracil that is in place of thymine. And a G>C mutation is expected when an AP site is generated from the excision of cytosine (Fig 2B). In absence of CDG expression, the deamination of cytosine would result in the generation of uracil leading to the G>C mutations after excision by Ung1. However, in *apn1Δ* background, the rate of G>C mutations is >40-fold lower than the rate of uracil-associated A>C and T>G mutations (S1 Table), indicating that the cytosine deamination in the yeast strains used in the study is negligible. Mutations resulting from the methylation of cytosine is also unlikely considering the previous report where methyl-cytosine was not detected in yeast genomic DNA [36].

We expressed CDG in the *apn1Δ ung1Δ* or *apn1Δ rad14Δ ung1Δ* strain backgrounds, where the endogenous *UNG1* is deleted so that all uracil- or cytosine-associated mutations are resulting from the activity of ectopically expressed CDG. The rates of uracil-dependent, A>C and T>G mutations as well as cytosine-dependent, G>C mutations were determined under the high (no DOX) and low (+DOX) transcription conditions to determine the effect of transcription on these mutations. Since the number of cytosine residues in DNA should not be affected by the level of transcription, the rate of mutations caused by the AP sites generated by the cytosine excision (G>C mutations) should not change whether under the high or low transcription conditions, unless the enzymatic efficiency of cytosine excision by CDG is affected by activated transcription. When CDG was expressed in the *apn1Δ ung1Δ* strain, the overall mutation rate was ~7-fold higher under the high transcription conditions than under the low transcription conditions (Fig 2C and S2 Table). The mutation spectra showed a ~10-fold increase in the rate of uracil-dependent, A>C and T>G mutations but only a ~1.7 -fold increase in the rate of the cytosine-dependent, G>C mutations (Fig 2D and 2E). In a BER/NER- deficient, *apn1Δ rad14Δ ung1Δ* strain, the CDG expression under high transcription conditions led to ~10- and ~ 2.5-fold increases in the rates of uracil- and cytosine-dependent mutations, respectively, compared to the CDG expression under low transcription conditions. Overall, the active transcription resulted in the significantly greater increase in the uracil-associated mutations compared to the cytosine-associated mutations. Altogether, these data suggest that the elevated glycosylase efficiency is only a minor factor contributing to the transcription-dependent elevation in the uracil-associated mutations.

### CDG glycosylase activity in the absence of Topoisomerase 1 or RNase Hs

**Fig 3:**
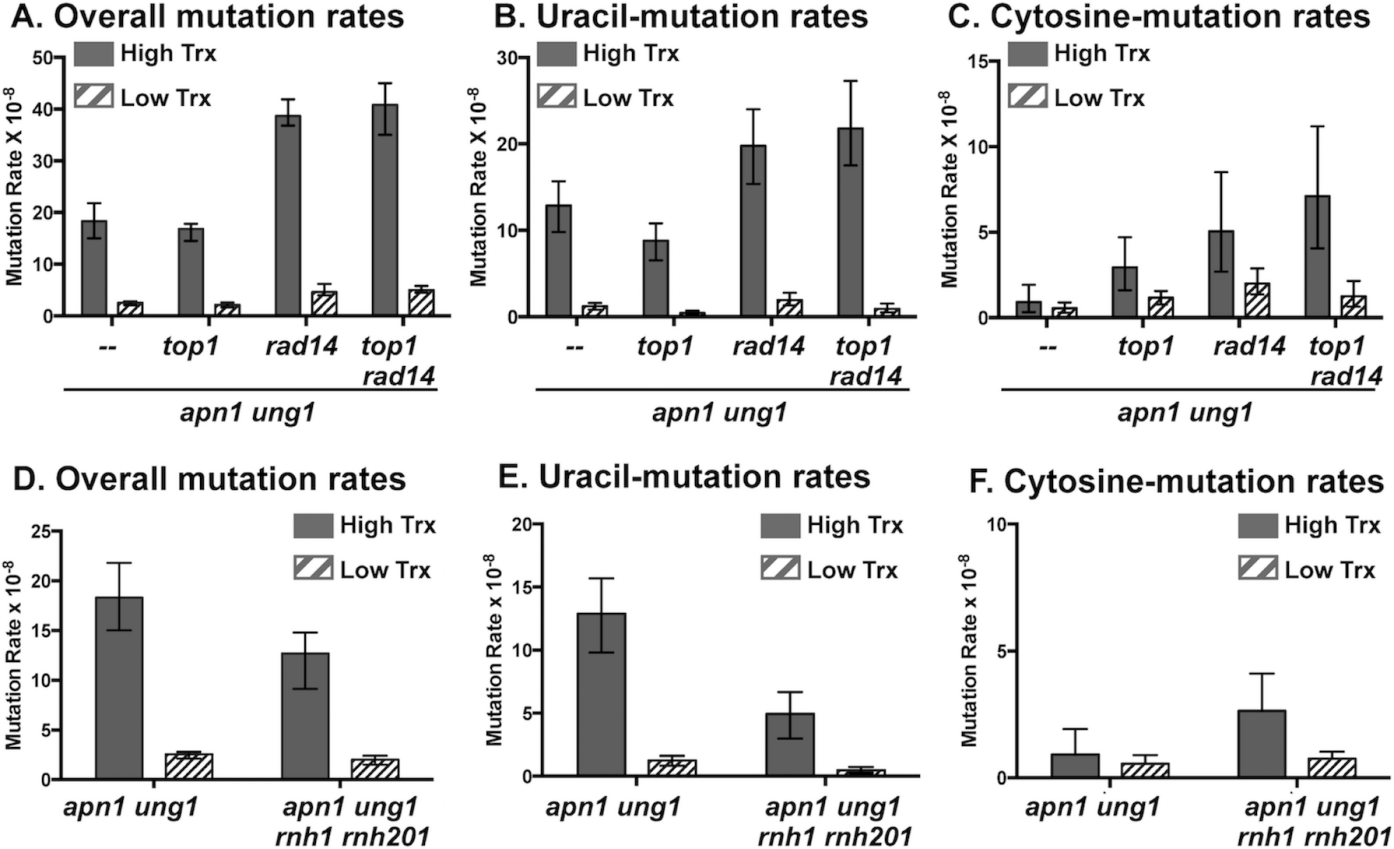
The effect of CDG expression on uracil- and cytosine-derived mutations in the absence of Topoisomerase 1 or RNase Hs. **A)** and **D)** Overall mutation rates in yeast strains with CDG glycosylase-expressing plasmid in under high-transcription (no doxycycline) or low-transcription (doxycyline added) conditions. Error bars represents 95% confidence intervals. **B), C), E) and F)** Rates of the uracil-dependent A>C and T>G mutations and the cytosine-dependent G>C mutations. The numerical values of the median mutation frequencies and the confidence intervals represented as graphs in this figure are listed in S2 Table. The mutation rates in **A)**, **B),** and **C)** are calculated from 24 independent cultures and the mutation rates in **D)**, **E)**, and **F)** are calculated from 12 independent cultures.

To further probe the effect of transcription on the activity of glycosylases, we repeated the CDG expression in *apn1Δ top1Δ ung1Δ* and *apn1Δ rad14Δ top1Δ ung1Δ* strains and determined the mutation rates under the high and low transcription conditions. Topoisomerase 1 (Top1) functions to relieve topological stress, including the transcription-associated supercoils, by creating transient strand breaks and then rejoining DNA strands [37]. Deletion of *TOP1* leads to an accumulation of negative helical stress, particularly in the highly transcribed areas. Upon CDG expression in the cells of *apn1Δ top1Δ ung1Δ* background, the rate of overall mutation at the *pTET-lys2-TAG* reporter was 7.9-fold higher under high transcription conditions than under low transcription conditions (Fig 3A). This is comparable to the 7.1-fold difference between the rates of CDG-induced mutations under high and low transcription conditions in *apn1Δ ung1Δ* background. Additionally, we sequenced the *pTET-lys2-TAG* allele in the Lys+ revertants to identify the specific nucleotide substitutions. In *apn1Δ top1Δ ung1Δ* background, CDG-expression under the high transcription conditions resulted in 16.3- and 27.6-fold increases in A>C and T>G mutations, respectively, compared to the CDG-induced mutations occurring under low transcription conditions (Fig 3B and 3C and S2 Table). These are substantially higher than the high-transcription associated elevation in A>C and T>G mutations observed in *apn1Δ ung1Δ* background, which were 4.2- and 11.2-fold increases, respectively (S2 Table). Additionally, when comparing the CDG-induced mutations, the transcription-dependent fold elevation (high/low) of A>C, T>G, and C>G mutations were significantly higher in *apn1Δ rad14Δ top1Δ ung1Δ* than in *apn1Δ rad14Δ ung1Δ*. As illustrated in Fig 2A, the uracil-derived A>C and T>G mutations result from the excision of uracil present on the transcribed (bottom) strand and the non-transcribed (top) strand, respectively. Since *TOP1* deletion resulted in the higher transcription-dependent elevation of both A>C and T>G mutations, the overall DNA topology changes in the Top1-deficient cells appear to increase the access of glycosylase to the uracil residues regardless of whether they are located on the transcribed or non-transcribed strand.

To determine whether uracil residues in the single-stranded DNA are more easily accessed by DNA glycosylases, we determined the rates of uracil- and cytosine-dependent mutations in the *apn1Δ rnh1Δ rnh201Δ ung1Δ* strain following CDG expression. *RNH1* and *RNH201* encode the RNaseH enzymes that degrade RNA hybridized to DNA [38]. During transcription, RNA-DNA hybrids form when the nascent RNA anneals to the template, transcribed DNA strand leaving the non-transcribed DNA strand single-stranded. In the absence of RNaseH enzymes, the transcription-associated RNA-DNA hybrids persist and accumulate to form “R-loops” [13]. In *apn1Δ rnh1Δ rnh201Δ ung1Δ* strain backgrounds, the rate of overall mutations induced by the CDG-expression at the *pTET-lys2-TAG* reporter under the high transcription conditions was not significantly greater than the rate in *apn1Δ ung1Δ* (Fig 3D). And the transcription-dependent increase in the rates of A>C or C>G mutations was not significantly different in these two backgrounds (Fig 3E and 3F and S2 Table). However, the transcription-dependent fold-increase (high/low) in the rate of T>G mutation was 39.1 in *apn1Δ rnh1Δ rnh201Δ ung1Δ* compared to 11.2 in *apn1Δ ung1Δ* (S2 Table). The greater effect of the disruption of RNase H enzymes on the T>G mutations relative to the nominal effect it had on the A>C or C>G mutations indicate that CDG has a greater access to the target (U or C) on the single-stranded non-transcribed strand of DNA.

### The long-amplicon qPCR experimental strategy to quantify uracil residues in DNA

**Fig 4:**
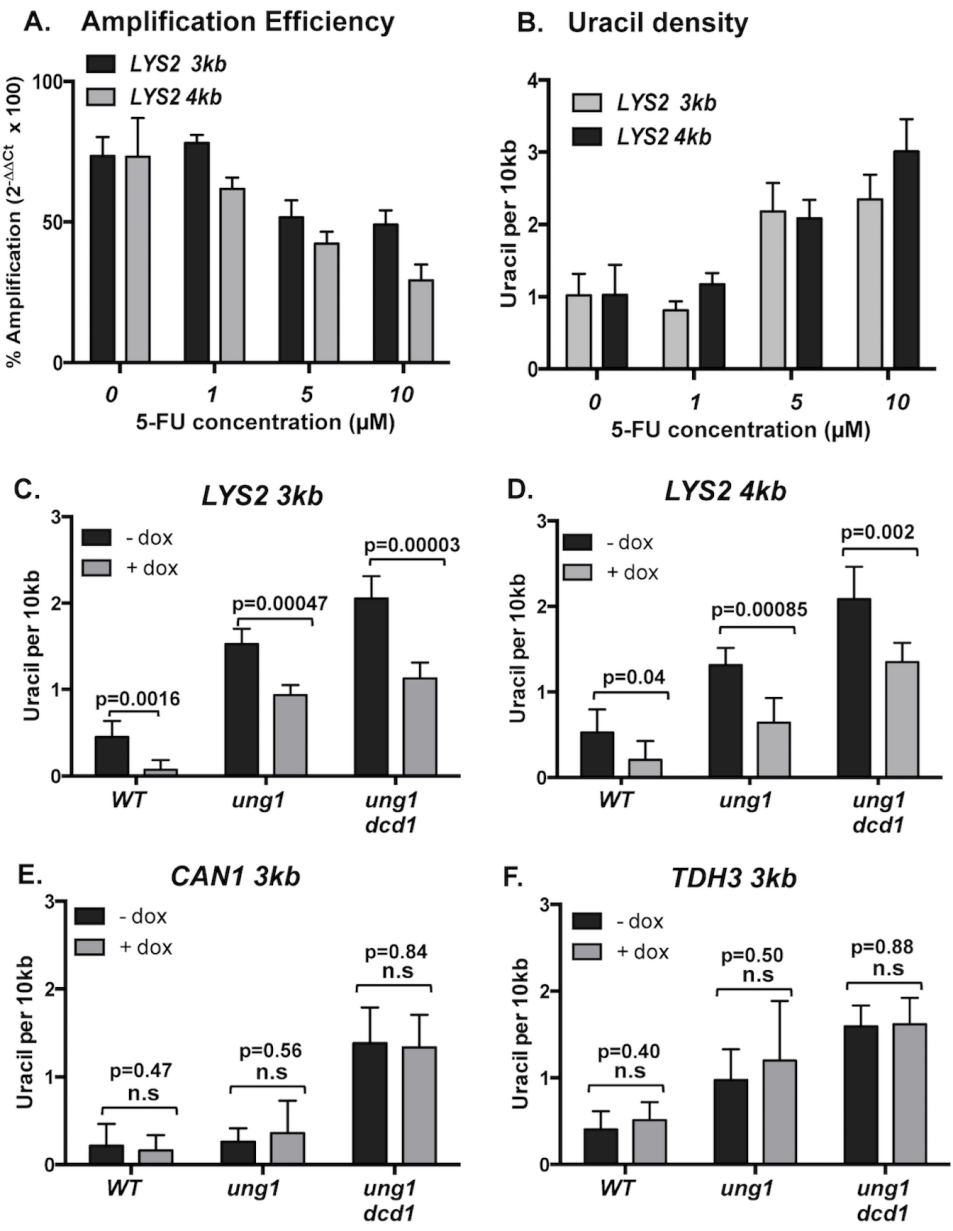
Uracil-density measured by the long amplicon qPCR approach. **A)** Relative percent amplification of the genomic DNA samples from the yeast cells treated with the indicated concentration of 5-FU for 24 hrs. **B)** The uracil-density of the genomic DNA samples calculated from the relative percent amplification shown in **A).** See MATERIALS AND METHODS for the equation used for the calculation. **C)–F),** the genomic DNA samples isolated from the indicated yeast strains grown in the absence (-dox) or presence (+dox) of 2 μg/mL doxycycline in the media were used for qPCR and the calculation of uracil-density. **C)** Uracil density at *LYS2* calculated from qPCR using “LYS2 3 kb” primers. **D)** Uracil density at *LYS2* calculated from qPCR using “LYS2 4 kb” primers. **E)** Uracil density at *CAN1* calculated from qPCR using “CAN1 3 kb” primers. **F)** Uracil density at *TDH3* calculated from qPCR using “TDH3 3 kb” primers. For **A) to F),** error bars represent standard deviation (*p-*values were calculated using the unpaired student t-test; n.s—not significant) and all measurements are from N = 6. The numerical data represented as graphs in this figure are listed in S3 Table.

To determine whether there is a correlation between the level of transcription and the uracil content in DNA, we sought to measure the density of uracil in DNA at specific genomic sites using the long-amplicon quantitative PCR approach. This method, which measures the reduction in the amplification efficiency resulting from the polymerase-blocking damage to the template DNA, was previously used to quantify the damage in the mitochondrial DNA of various vertebrate species [39] and the uracil residues at the mouse immunoglobulin loci in B lymphocytes [40]. Genomic DNA isolated from yeast cells lacking the endogenous Ung1 was treated *in vitro* with recombinant UDG to create AP sites specifically at the uracil residues. The resulting AP site was then converted into a single-strand break by the Endo VIII-treatment. The presence of uracil is measured as the relative loss of qPCR signal or the relative reduction in the amplification efficiency when the UDG/Endo VIII-treated DNA is amplified compared to when an untreated DNA sample is amplified. First, to validate our strategy, we measured uracil content in *ung1Δ* yeast cells treated with 0, 1, 5 and 10 μM 5-fluorouracil (5-FU), an inhibitor of TS that leads to an increase in the cellular dUTP pool and the accumulation of uracil residues in DNA. The DNA samples purified before or after UDG/Endo VIII-treatment were used as the template in qPCR reactions with *LYS2* primers targeting ~100 bp, 3 kb, and 4 kb regions (Fig 4A and S1 Table). The Ct values from the “LYS2 100 bp” primers were used as normalizing controls, under the assumption that it is highly unlikely there is a significant number of the polymerase-blocking lesions within the approximately 100 bp region targeted by these primers. For the DNA sample from cells treated with no or 1μM 5-FU, the amplification of the UDG/EndoVIII-treated DNA were about 75% of the untreated DNA. For the samples treated with 5 and 10 μM 5-FU, there was a dose-dependent reduction in the amplification efficiency compared to that of untreated samples (Fig 4A). As expected, the amplification of the 4 kb amplicon (“LYS2 4kb” primer set) had a significantly reduced amplification in comparison to the 3 kb amplicon (“LYS2 3kb” primer set) since the larger amplicon size increases the likelihood of a lesion being encountered during PCR. We used a Poisson equation to further estimate the average uracil frequency at the *LYS2* locus from the long-amplicon amplification efficiency and observed that the uracil frequency is significantly elevated when treated with higher concentrations of 5-FU (Fig 4B). The frequency of uracil in DNA was about 1 per 10 kb in cells treated with 0 or 1 μM 5-FU and was elevated to >2 per 10 kb with the treatment of 5 or 10 μM 5-FU. Overall, these results show that the long-amplicon qPCR strategy can be used to quantitatively measure the uracil frequency in yeast genomic DNA.

### Uracil residues enhanced at highly transcribed genomic loci

We extended the long amplicon qPCR method to determine the endogenous uracil levels at several genomic loci. In order to first determine the level of transcription of the genes where we measure the uracil frequency, we isolated RNA from *ung1Δ* and *ung1Δ dcd1Δ* strains grown in the presence or absence of doxycycline. *DCD1* is a gene that encodes a deoxycytidylate deaminase (Dcd1), which converts dCMP to dUMP, a substrate for the dTTP production [32]. The deletion of *DCD1* increases the [dUTP]/[dTTP] ratio, leading to the increased incorporation of uracil into DNA. As expected from the elevated level of uracil-incorporation into DNA, the deletion of *DCD1* in *apn1Δ* strain led to a two-fold increase in the rate of mutation at the *pTET-lys2-TAA* reporter under high transcription conditions (S1A Fig). Following mRNA extraction from *ung1Δ* and *ung1Δ dcd1Δ* strains, we performed RT-qPCR to determine the expression levels of *pTET-lys2-TAA*, *CAN1* and *TDH3* in the presence and absence of doxycycline. While the expression levels of *CAN1* and *TDH3* genes, which are not regulated by the *pTET* promoter, was not affected by the presence of doxycycline, the level of *pTET-lys2-TAA* transcripts from no DOX samples was ~170-fold higher than that from +DOX samples (S1B Fig).

For the long-amplicon qPCR analysis, DNA samples were prepared from *WT*, *ung1Δ*, and *ung1Δ dcd1Δ* strains grown in the presence and absence of doxycycline. Under the conditions where the transcription of *pTET-lys2-TAA* is repressed (+ DOX), the density of uracil in DNA as inferred from the amplification carried out with the “LYS2 3kb” primers were 0.08, 0.93, and 1.1 per 10 kb in *WT*, *ung1Δ*, and *ung1Δ dcd1Δ*, respectively (Fig 4C). Under high transcription conditions (no DOX), the uracil-density was significantly higher in all three strain backgrounds with 0.45, 1.5, and 2.1 per 10 kb in *WT*, *ung1Δ*, and *ung1Δ dcd1Δ* strains, respectively. In WT cells, the activity of endogenous Ung1 accounts for the relatively low level of uracil in DNA in WT as determined by the long-amplicon PCR approach. When “LYS2 4kb” primers were used for the analyses on the same DNA samples, the uracil-densities calculated were 0.53, 1.3, and 2.1 per 10 kb in *WT*, *ung1Δ*, and *ung1Δ dcd1Δ*, respectively, under high transcription conditions and statistically the same as those calculated using “LYS2 3kb” primers (Fig 4D). For the *CAN1* or *TDH3* loci, a set of primers targeting a ~3 kb region encompassing each gene was used in the long amplicon PCR with another set of primers targeting a ~100 bp region within each gene as the control PCR reactions (S1C Fig). In a manner similar to the *pTET-lys2-TAA*, the density of the uracil in DNA were highest in the *ung1Δ dcd1Δ* background and lowest in WT background (Fig 4E and 4F). Unlike the *pTET-lys2-TAA*, the addition of doxycycline did not affect the uracil density at *CAN1* or *TDH3* loci. In *ung1Δ* background, the uracil density was about 3-fold lower at *CAN1* than *TDH3*. However, in *ung1Δ dcd1Δ* background, the uracil density derived by the long-amplicon PCR approach at *TDH3* and *CAN1* were not significantly different. In both *ung1Δ* and *ung1Δ dcd1Δ* backgrounds, *TDH3* is transcribed at ~100-fold higher rate than *CAN1* (S1B Fig).

### Uracil-dependent mutations increase when repair synthesis is induced by 4NQO or CPT

**Fig 5:**
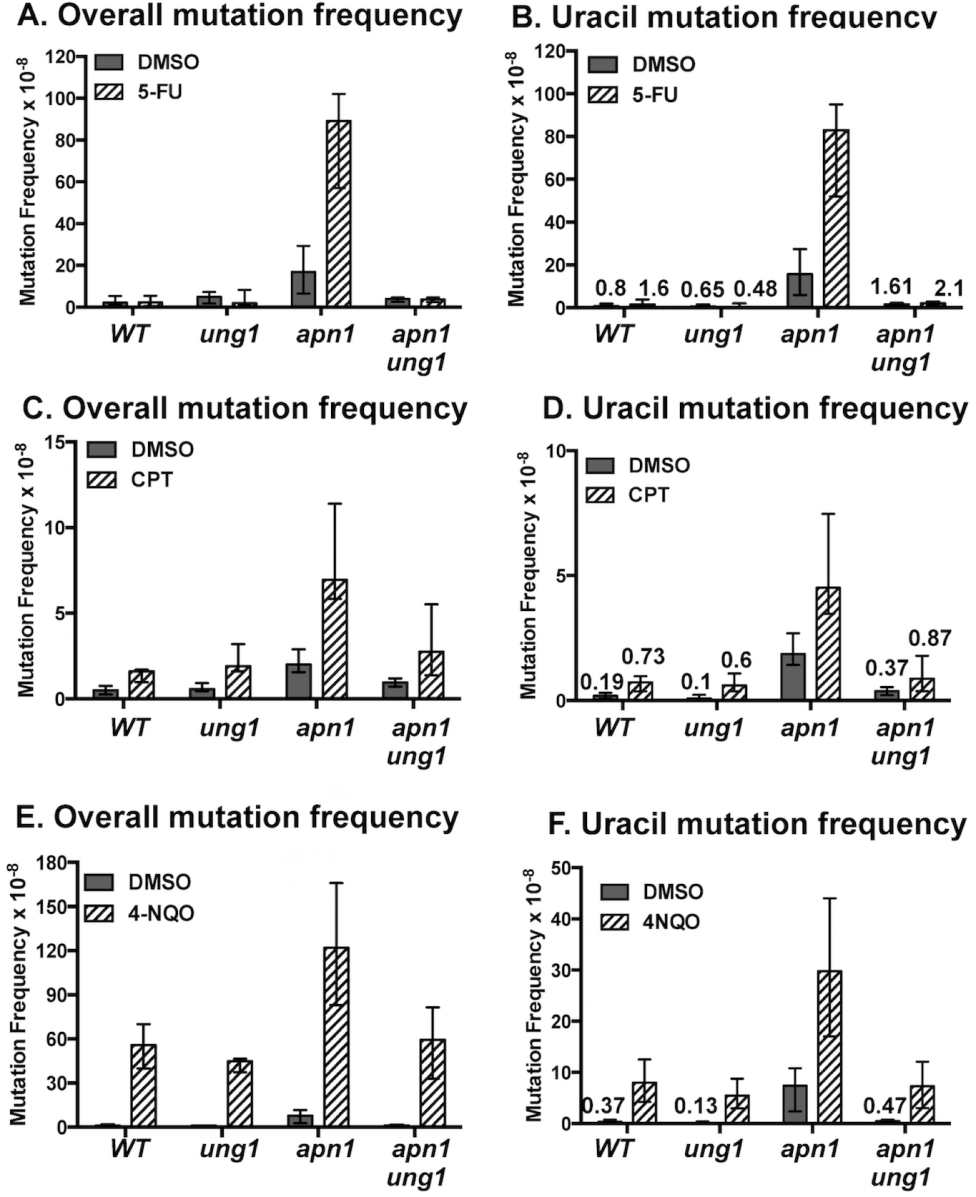
Mutation frequencies following the 5-FU-, 4NQO-, and CPT-treatment. **A)**, **C),** and **E)** The frequencies of overall Lys+ mutations following treatments with 5-FU (10 μM), CPT (100 μM), or 4NQO (0.2 μg/mL), respectively, for 24 hrs. **B), D),** and **F)** The frequencies of the uracil-dependent A>C and T>G mutations following treatments with 5-FU, CPT and 4NQO, respectively. Error bars indicate 95% confidence intervals. The number of cultures used to determine the frequencies of mutations and the numerical values of the median mutation frequencies and the confidence intervals represented as graphs in this figure are listed in S4 Table.

We postulate that spontaneous DNA damage associated with the active transcription lead to cycles of unscheduled DNA synthesis (UDS) leading to the increase in uracil-associated mutations. In order to test this hypothesis, we induced UDS using three different types of DNA damaging agents and calculated the frequency of uracil-dependent mutations. First, we treated the cells with 5-fluorouracil (5-FU), which imbalances the [dUTP]/[dTTP] ratio and thus enhance the incorporation of uracil residues in DNA. The frequency of mutations at the reporter was determined in *WT*, *ung1Δ*, *apn1Δ*, and *apn1Δ ung1Δ* background cells treated with 10μM 5-FU. Compared to the DMSO control, the 5-FU treatment led to a remarkable elevation in the rate of *pTET-lys2-TAA* mutations in the BER-deficient *apn1Δ* strain but not in the BER-proficient *WT* or *ung1Δ* strain. As would be expected of mutations arising from the uracil-derived AP sites, the rate of mutations was far reduced in the uracil-excision incapacitated *apn1Δ ung1Δ* compared to that in *apn1Δ* (Fig 5A). When the Lys+ mutants were sequenced, the majority (67/72) in *apn1Δ* strain were A>C or T>G, as expected of uracil-associated mutations (Fig 5A and 5B).

In order to determine whether the repair induced by a broad spectrum of DNA damage can increase the uracil-incorporation into DNA and the consequent uracil-derived mutations, we measured the frequency of the *pTET-lys2-TAA* mutations in yeast cells treated with two DNA damaging agents without previously reported effect on the dUTP/dTTP metabolic pathway -camptothecin (CPT) and 4-nitroquinoline 1-oxide (4NQO). CPT is a Top1 inhibitor that traps Top1-DNA cleavage complex and is known to elevate recombination and copy number variations in the eukaryotic genomes [41, 42]. Following CPT-treatment, the overall mutations were elevated by ~3 folds in *WT*, *ung1Δ*, *apn1Δ*, and *apn1Δ ung1Δ* backgrounds (Fig 5C). The mutation spectra and the frequency of the specific type of mutations were determined by sequencing the *pTET-lys2-TAA* reporter in Lys+ revertants. In *apn1Δ* cells, the increase in the rate of A>C and T>G mutations due to CPT-treatment was statistically significant with the 95% confidence intervals not overlapping (Fig 5D). In *apn1Δ ung1Δ* cells, the frequency of A>C and T>G mutations in the CPT-treated samples was slightly higher than the untreated control but the difference was not statistically significant. Also, A>C and T>G mutations in the CPT-treated *apn1Δ ung1Δ* were significantly reduced compared to those in *apn1Δ*, indicating that the mutations resulting from Ung1-mediated excision of uracil in DNA do occur at a significant level upon CPT-treatment.

4NQO is a mutagenic heterocyclic chemical that forms covalent bulky adducts to dG or dA, which are predominantly repaired by NER [43]. Following treatments with 4NQO, the overall mutation frequency at the *pTET-lys2-TAA* reporter was elevated by ~15- to 50-fold in *WT*, *ung1Δ*, *apn1Δ*, and *apn1Δ ung1Δ* backgrounds (Fig 5E). The mutation spectra revealed that a majority of the mutations elevated by the 4NQO-treatment were A:T > T:A transversions (S2 Fig), the type of mutations that had previously been associated with 4NQO [44]. The uracil-dependent mutations (A>C and T>G) were significantly elevated only in the *apn1Δ* cells treated with 4NQO (Fig 5F). Similar to the observation in the CPT-treated cells, the frequency of 4NQO-induced A>C and T>G mutations in the *apn1Δ ung1Δ* background was significantly lower than that in *apn1Δ*, indicating that uracil-incorporation into DNA and the resulting mutations also occur at a significant level following the 4NQO-treatment.

### The level of uracil in DNA increases when repair synthesis is induced using 4NQO

**Fig 6:**
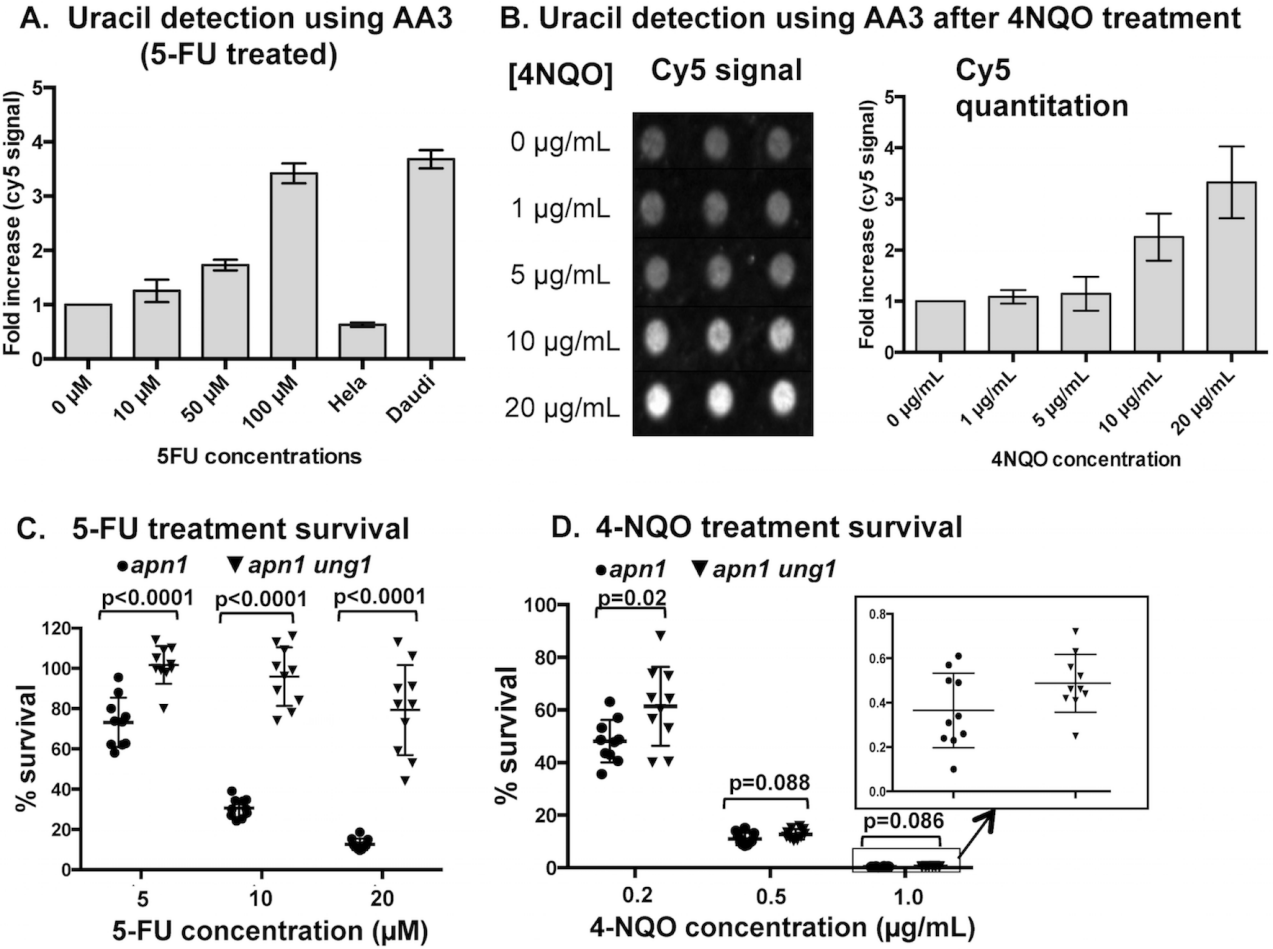
Uracil residues in the DNA following the 4NQO-treatment. **A)** Quantification of uracil residues in DNA from *ung1Δ* yeast cells treated with the indicated concentrations of 5-FU for 24 hrs. The genomic DNA samples from untreated Hela and Daudi are used as negative and positive controls, respectively. All measurements are from N = 6. **B)** Quantification of uracil residues in DNA from yeast cells treated with the indicated concentrations of 4NQO. Three replicates are shown in the figure for each 4NQO concentrations. **Left**- Cy5 signal from AA3-labeled DNA dot-blotted on a nylon membrane. **Right**- Quantification of the cy5 signal shown on the left. Cy5 quantity is represented as relative to untreated (0μg/mL) sample. The numerical data represented as graphs are listed in S5 Table. Error bars indicate standard deviations and all measurements are from N = 6. Survival after growth in **C)** the 5-FU- or **D)** 4NQO-supplemented liquid culture, represented as the relative percentage compared to the untreated cells (*p-*values were calculated using the unpaired student t-test). N = 10.

In order to quantify the uracil residues present in the genomic DNA, we used an AP-reactive alkoxyamine compound AA3 [45]. This compound contains the alkyne group, through which a variety of compounds can be attached *via* click chemistry. Our approach to detect uracil-residues in DNA was first to label the uracil-derived AP sites with AA3 and then to attach the fluorescent dye cyanine 5 (Cy5) to AA3-AP conjugates (S3 Fig). To demonstrate its efficacy, we applied the approach to the *ung1Δ* cells treated with various concentration of 5-FU and observed a dose-dependent increase in the Cy5 signal when yeast cells were treated with 10, 50, and 100 μM 5-FU (Fig 6A). Genomic DNA isolated from Hela cells and the AID-expressing Daudi cells was used as negative and positive controls, respectively. Daudi is a B-cell lymphoma cell line with highly elevated level of uracil in DNA resulting from the overexpression of the APOBEC family of cytosine deaminases [46, 47]. While there was no significant difference between the level of uracil in yeast cells without 5-FU treatment and that in Hela cells, the level of uracil in DNA in yeast cells treated with 100 μM 5-FU increased to the level comparable to that in Daudi cells (Fig 6A). Using the same AA3-Cy5 labeling approach, we measured the level of uracil in DNA in yeast cells treated with 1, 5, 10, and 20 μg/mL 4NQO. The levels of uracil detected in cells treated with 10 or 20 μg/mL 4NQO, but not with 1 or 5 μg/mL 4NQO, were significantly elevated compared to the untreated sample (Fig 6B).

### Sensitivity to 4NQO is partially reduced by UNG1 deletion

To further show that the uracil-incorporation into DNA is a significant component of the repair synthesis associated with 4NQO treatment, we tested whether uracil in DNA is relevant to the cytotoxicity of 4NQO. The main cytotoxic lesion of 5-FU treatment is the AP sites derived from the uracil incorporated into the genomic DNA. When treated with 5-FU, yeast cells in the BER deficient *apn1Δ* backgrounds are highly sensitive (Fig 6C). But the 5-FU sensitivity is very significantly reduced in *apn1Δ ung1Δ* background, where uracil in DNA cannot be removed to create the toxic AP sites. If 4NQO treatment elevates the level of uracil in DNA, *apn1Δ ung1Δ* strains would have a reduced level of sensitivity to 4NQO in comparison to *apn1Δ* strains. We calculated the number of surviving colony-forming cells after culturing them in the liquid media with or without 0.2, 0.5, or 1 μg/mL 4NQO and observed a slight survival advantage in *apn1Δ ung1Δ* compared to *apn1Δ*, although only the 2 μg/mL 4NQO concentration elicited a statistically significant increase (Fig 6D). Together, these results support our hypothesis that uracil incorporation into DNA occurs at a significant level when DNA repair synthesis is induced.

### Dut1 is cell-cycle regulated in yeast

We hypothesized that the transcription-associated increase in the density of uracil in DNA as determined by the long-amplicon qPCR or the CDG expression experiments above is due to the incorporation of uracil into DNA during unscheduled DNA synthesis (UDS) that can occur outside of the genome duplication in S phase. It has been shown in mammalian cells and plants that *DUT1*, a gene that encodes for dUTPase, is cell-cycle regulated with its highest expression in S-phase [48, 49]. In yeast, a large-scale high through-put analysis previously has shown that the dUTPase-encoding *DUT1* gene begins to be upregulated in late G1 phase ensuring that dUTP levels are kept low during replication [50]. Conversely, DNA synthesis occurring outside of S phase (i.e. G1 and G2) will be subject to the dNTP pool with the relatively higher dUTP levels. To confirm the cell-cycle regulated *DUT1* expression in yeast, we arrested cells in G1 using the mating pheromone α-factor and collected cells every 15 minutes after release for the RNA isolation and qRT-PCR. The expression level of the histone H2-encoding *HTA2* gene, which was previously shown to be upregulated during S phase, was determined as a control [51]. The expression levels of both *DUT1 and HTA2* genes were highest at 45 mins after the release from α-factor and declined to the lowest point at 75 mins after the release indicating that the *DUT1* expression is cell-cycle regulated in a manner similar to the *HTA2* gene (S4A Fig).

### Overexpression of Dut1 in G1 or G2 significantly reduces the transcription-associated mutations

We previously reported that the plasmid-mediated overexpression of *DUT1* from the galactose-inducible *pGAL* promoter can greatly reduce the uracil-associated mutations at the *pTET-lys2-TAA* reporter [18], indicating that the cellular [dUTP] is a critical determinant in the transcription-associated uracil-dependent mutations. When induced by the addition of galactose to the media, the *pGAL*-regulated genes are highly expressed regardless of the cell cycle. To test whether the uracil-incorporation into DNA during G1 and G2 phases is a significant contributor to the transcription-associated uracil-dependent mutations, we modulated [dUTP] in G1, S and G2 phases by the cell-cycle specific overexpression of *DUT1* gene and measured the effect on the mutation rate at the *pTET-lys2-TAA* reporter. For the cell-cycle specific expression of *DUT1* in G1, S, or G2 phase, we replaced the *pGAL* with the promoters of *CLN2*, *HHO1*, or *CLB2* genes, respectively [14, 52]. In order to reduce the protein half-life and thereby ensure cell-cycle specific presence of the overexpressed Dut1 protein, we added a protein destabilization domain (PEST) to the plasmid constructs [53]. We expressed *DUT1-PEST* from these plasmid constructs in yeast, isolated mRNA from asynchronous cells and performed qRT-PCR to determine the expression level. *DUT1* was expressed 38-, 64-, and 25-fold higher than the endogenous level from the G1, S, and G2 constructs, respectively. For all three constructs, the level of the overexpressed *DUT1* mRNA was substantially lower than the *DUT1* expression from *pGAL*-construct, which was 412-fold higher than the endogenous level (S4B Fig). In order to confirm that *DUT1* is expressed in the cell cycle-specific manner from these promoters, we performed qRT-PCR with RNA samples isolated every 20 minutes after the release of cells arrested at G1 with α-factor. The *DUT1* mRNA expression was highest at 100, 40, and ~60–100 mins after release from α-factor (S4C, S4D, and S4E Fig). The S-phase time point under this specific condition was determined to be 60 mins after the release from the α-factor by analyzing the mRNA level of *HTA2* gene.

**Fig 7:**
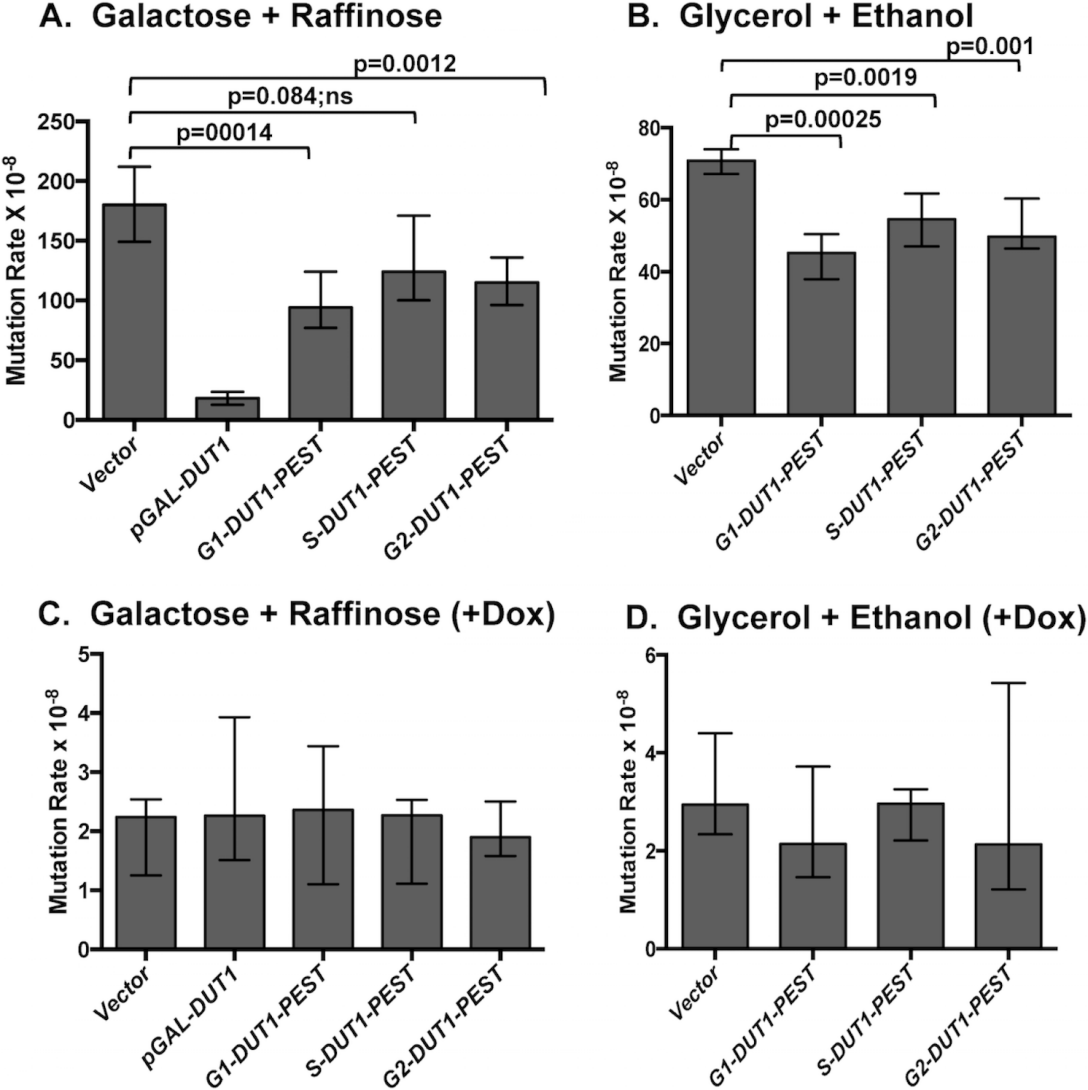
The rates of mutation in cells overexpressing Dut1 from the cell-cycle regulated promoters. The rates of Lys+ reversion mutations of the *apn1Δ ntg1Δ ntg2Δ* strain transformed with plasmids expressing *DUT1* from *pGAL*, p*CLN2 (G1)*, p*HHO1 (S)*, or p*CLB2* (G2). The growth conditions were in rich media supplemented with galactose and raffinose **(A)**, glycerol and ethanol **(B),** galactose and raffinose plus doxycycline **(C),** or glycerol and ethanol plus doxycycline **(D)**. The error bars indicate 95% confidence intervals. (*p-*values were calculated using the unpaired student t-test). The number of cultures used to determine the rates of mutations and the numerical values of the median mutation rates and the confidence intervals represented as graphs in this figure are listed in S6 Table.

We transformed the BER-deficient, *apn1Δ ntg1Δ ntg2Δ* cells with the plasmids containing p*CLN2 (G1-)*, p*HHO1 (S-)*, or p*CLB2 (G2)-*constructs and calculated the mutation rates at the *pTET-lys2-TAA* reporter. We first carried out the fluctuation analysis for the determination of mutation rates in media supplemented with galactose and raffinose under high transcription conditions in order to compare the effect of Dut1 expression from p*CLN2*, p*HHO1*, or p*CLB2* to its expression from the previously studied *pGAL*-construct. While the *pGAL*-construct resulted in ~10-fold decrease in the rate of mutations compared to the vector-only control under these conditions, the G1-specific, p*CLN2*-construct led to a ~ 2-fold reduction in mutation rates in cells (Fig 7A). Although the G2-specific, p*CLB2-*construct led to a <2-fold reduction, the mutation rate in the cells with this construct was significantly different from that in the cells with vector alone. For the cells with S-specific *pHHO1*-construct, there was no significant reduction in the mutation rate compared to the vector control. When the fluctuation analysis was repeated in the media supplemented with glycerol and ethanol, the G1-, S-, and G2-specific constructs all resulted in <2-fold, but statistically significant, reductions in the rates of mutation at the *pTET-lys2-TAA* reporter compared to the vector control (Fig 7B). When the transcription of the *pTET-lys2-TAA* was repressed by adding doxycycline, the rates of mutation was unchanged with the Dut1-overexpression from the G1-, S-, or G2-specific promoters or from the *pGAL* promoter (Fig 7C and 7D). These results suggest that the shift in the free dUTP pool affects the uracil-composition and the uracil-associated mutations more substantially at highly transcribed genes.

## Discussion

High levels of transcription have previously been implicated as a major source of genomic instability in various organisms (reviewed in [4, 54, 55]. In yeast, when a reporter construct with the tetracycline-regulatable promoter (*pTET*) was used to determine the rate of mutations at several different levels of transcription, a linear and proportional relationship between the level of transcription and the rate of mutation was observed [56]. Subsequent studies indicated that a majority of these mutations were derived from unrepaired abasic sites [18]. When BER is disabled, as in *apn1Δ* or *apn1Δ ntg1Δ ntg2Δ* strains, there was a unique elevation in specific types of mutations. Namely, when the mutations at the *pTET-lys2-TAA* allele was studied, TAA to GAA, TCA, or TAC mutations were elevated by ~200- and 500-folds in *apn1Δ* or *apn1Δ ntg1Δ ntg2Δ* strains, respectively [19]. The highly elevated rates of these T>G and A>C mutations were dramatically reduced by the disruption of the uracil DNA glycosylase Ung1 or by the overexpression of the dUTPase Dut1, indicating that these types of mutation are originating from uracil in DNA. There are two distinct ways by which the non-canonical uracil residues appear in DNA; by the deamination of cytosine residues present in DNA or by the incorporation into DNA by DNA polymerase utilizing dUTP in place of dTTP. The location of mutations at T:A or A:T pairs suggests the latter route of uracil appearance in DNA. Further genetic studies showed that AP sites generated by the excision of uracil by Ung1 is bypassed by the TLS polymerases Rev1 and Polζ to bring about the T>G and A>C mutations [57]. The most remarkable finding about these uracil-derived T>G and A>C mutations was that they are almost completely suppressed when the transcription of the *pTET-lys2-TAA* mutation reporter is repressed by the addition of doxycycline. This transcription-dependent elevation of mutations originating from uracil residues in DNA led to the hypothesis that the chemical composition of the DNA can be changed to include a higher number of uracil residues when actively transcribed.

We tested the hypothesis of the transcription-dependent elevation of uracil residues in DNA by directly quantifying uracil residues at a defined genomic locus under high or low transcription conditions using the long-amplicon qPCR method. As we demonstrated with cells treated with various concentrations of 5-FU, the cellular balance between dUTP and dTTP concentrations is the key determinant of the density of uracil in DNA (Fig 4B). Therefore, we carried out the measurement of the endogenously present uracil residues in DNA in WT and *ung1Δ* strain backgrounds rather than in the *apn1Δ* or *apn1Δ rad14Δ* backgrounds where the severe defect in BER and/or NER pathways could possibly disrupt the regulation of dNTP pool. In *ung1Δ* strains, where uracil residues, once incorporated into the DNA, cannot be excised out, there was a statistically significant 2-fold difference between the densities of uracil detected at the *pTET-lys2-TAA* under high and low transcription conditions (Fig 4C and 4D). The disruption of Dcd1, a dCMP deaminase, moderately reduces the dTTP production, thereby resulting in imbalance in [dUTP] to [dTTP] ratio. Such a shift in [dUTP]/[dTTP] has been shown to elevate the uracil incorporation into DNA in previous reports [58] and the uracil-associated mutations in the current study (S1A Fig). Significant elevations of the uracil density at the *pTET-lys2-TAA* under both high and low transcription conditions were observed upon the deletion of *DCD1* gene (Fig 5). The densities of uracil at the *CAN1* and *TDH3* genes were also elevated by the disruption of Dcd1. These data again demonstrate that the difference in the uracil density calculated using the long-amplicon qPCR approach is critically dependent on the [dUTP]/[dTTP] balance and adequately reflects the change in the DNA composition. At *CAN1* and *TDH3* genes, the levels of uracil as well as the rates of transcription did not change when doxycycline was added to repress transcription from the *pTET* promoter (Figs 4 and S1B). There was a ~170-fold difference in the level of transcription at the *pTET-lys2-TAA* between high and low transcription conditions while the level of uracil is elevated by ~2-fold. At *TDH3*, which is transcribed at ~100-fold higher rate than *CAN1* according to the RT-qPCR analysis, the uracil density was detected to be ~3-fold higher than at *CAN1* in *ung1Δ* background, providing further corroboration for the transcription-dependent mechanism of uracil incorporation into DNA. However, when the uracil density at the *pTET-lys2-TAA* is compared to those at *CAN1* and *TDH3* genes, we observed that the transcription rate does not have a linear correlation with the level of uracil residues. Under the low transcription conditions, the *pTET-lys2-TAA* is transcribed at a considerably lower rate than the *CAN1* gene. However, under the same conditions, there was no statistical difference in the uracil densities at these two genomic sites. On the other hand, the uracil level at the *pTET-lys2-TAA* under the high transcription conditions was slightly higher than that at the *TDH3* gene although the latter is transcribed at about ~10-higher rate than the *pTET-lys2-TAA*. These discrepancies suggest that there might be factors additional to transcription that modulate the level of uracil-incorporation into DNA such as position of the replication fork, replication timing, and orientation of the transcription machinery.

In *apn1Δ* strain, the rate of uracil-derived mutations is elevated by ~20-fold when transcription from the *pTET* promoter is activated [18]. The approximately 2-fold difference in the uracil density cannot wholly account for the dramatic increase in the mutation rate. An alternative, but not mutually exclusive, explanation for the transcription-dependent elevation in the mutations arising from uracil in DNA is that transcription affects the activity of the glycosylase converting the mutation-neutral uracil residues into the mutagenic AP sites. In order to test this hypothesis, we studied the mutations induced by the glycosylase CDG, which excises undamaged cytosines in addition to uracil residues, at *the pTET-lys2-TAG* mutation reporter under high and low transcription conditions (Figs 2 and 3). If the base-excision by the glycosylase is not affected by the state of transcription, the rate of those mutations initiated by the excision of undamaged cytosine (G>C) should remain the same whether the transcription of *pTET-lys2-TAG* reporter is activated or repressed. In five different genetic backgrounds, the CDG expression led to significant transcription-dependent elevations of the uracil-dependent (A>C and T>G) mutations as expected from the greater uracil density under high transcription conditions. However, we also observed smaller but still significant transcription-dependent elevations in the rate of cytosine-dependent mutations. The fold-difference between the high and low transcription conditions was 1.7 and 2.5 in *apn1Δ ung1Δ* and *apn1Δ rad14Δ ung1Δ* backgrounds, respectively, and increased to 2.5 and 7.6 when *TOP1* gene was deleted from each of these strains (Figs 2E and 3C and S2 Table). Compared to the transcription-dependent elevation in the CDG-induced A>C and T>G mutations, which ranges from 10- to 23-fold, the elevation of CDG-induced mutations at cytosine residues is relatively small but still significant, indicating that the efficiency of glycosylase activity is somehow affected by transcription (Figs 2D and 3B and S2 Table).

For the uracil-associated A>C and T>G mutations, the transcription-dependent elevation is further augmented by ~2-fold when the topoisomerase I is disrupted, implicating the transcription-associated change in the local DNA topology as one of the major factors affecting the glycosylase activity (Fig 3B). When RNase Hs were disrupted as in *apn1Δ rnh1Δ rnh201Δ ung1Δ* strain, the cytosine-dependent mutations at *the pTET-lys2-TAG* were elevated by 3.5-fold when transcription was highly activated (Fig 3F). For the uracil-derived mutations, only the mutations originating from the excision of uracil located on the top, non-transcribed strand (i.e. T>G) were further elevated by the disruption of RNase Hs (S2 Table). The R-loop accumulation in the absence of RNase Hs affects the two DNA strands within the transcribed regions asymmetrically; the bottom, transcribed strand forms stable hybrid with the nascent RNA and the top, non-transcribed strand is left unpaired. This asymmetry is directly reflected on the specific elevation of T>G over A>C mutations at *the pTET-lys2-TAG* and corroborates the biochemical analysis where CDG was shown to excise uracil or cytosine from single-stranded oligonucleotide substrates ~10-fold more efficiently than from double-stranded substrates [34]. The accumulations of helical stress and single-stranded DNA patches associated with active transcription appear to be major factors in enhancing the activity of uracil DNA glycosylase, contributing to the elevated uracil-dependent mutations at highly transcribed genes.

With the long-amplicon qPCR and the analysis of CDG-induced mutations, an increase in the density of uracil upon activation of transcription has been demonstrated by two independent approaches (Figs 2, 3 and 4). The mechanism underlying such phenomena, however, is still not clear. One plausible explanation can be found in the previously reported evidence of transcription-induced endogenous DNA damage (reviewed in [55]). The topological changes and DNA strand-separation necessitated by transcription is also responsible for the elevated susceptibility to genotoxic agents and the consequent accumulation of base damage as well as promoting the formation of pathological RNA:DNA hybrids or R-loops. The highly transcribed areas of the genome are more prone to the replication fork stalls and collapse, which can be significantly aggravated at repetitive sequences where transcription facilitates the formation of non-B DNA structures. While the DNA polymerases utilizing dUTP in place of dTTP during replication can account for the stochastic presence of uracil throughout the genome, DNA synthesis associated with correcting the damage or resolving the stalled replication fork at highly transcribed regions provides additional opportunities to incorporate uracil into DNA and to affect the locus-specific elevation in uracil content. Whether these involve only the short patch DNA synthesis required during NER/BER-mediated repair of base damages or additionally involve the more extensive DNA synthesis occurring in the processes of homologous recombination or break induced replication is to be determined by further investigation.

In support of the model where the repair-synthesis increases the incidence of uracil incorporation into DNA, we demonstrated here that exogenously engendering damage to DNA with CPT and 4NQO, thereby inducing rounds of repair-dependent DNA synthesis, led to an accumulation of uracil specific mutations into DNA in the absence of BER at the *pTET-lys2-TAA* mutation reporter (Fig 5). In case of 4NQO-treated cell, we also showed the uracil-accumulation in the genome by chemically probing for the uracil-derived AP sites (Fig 6B). Another important implication of these experiments is that uracil in DNA could be a significant factor in not only the mutations but also the cytotoxicity induced by various DNA damaging chemicals not specifically targeting the pyrimidine biosynthesis pathway. For the TS-targeting drug 5-FU, the main cytotoxic mechanism involves the AP sites generated from the highly frequent uracil residues incorporated into DNA due to the imbalance in [dUTP]/[dTTP] ratio [59]. The yeast cells of *apn1Δ* background with the severely compromised BER pathway and the inability to efficiently repair AP lesions are acutely sensitive to 5-FU. However, the *apn1Δ* cells become highly resistant to 5-FU when *UNG1* is deleted so that the uracil-to-AP conversion cannot occur (Fig 6C). We showed that *UNG1*-deletion can also reduce the cell sensitivity to 4-NQO treatment at a low drug concentration (Fig 6D).

**Fig 8:**
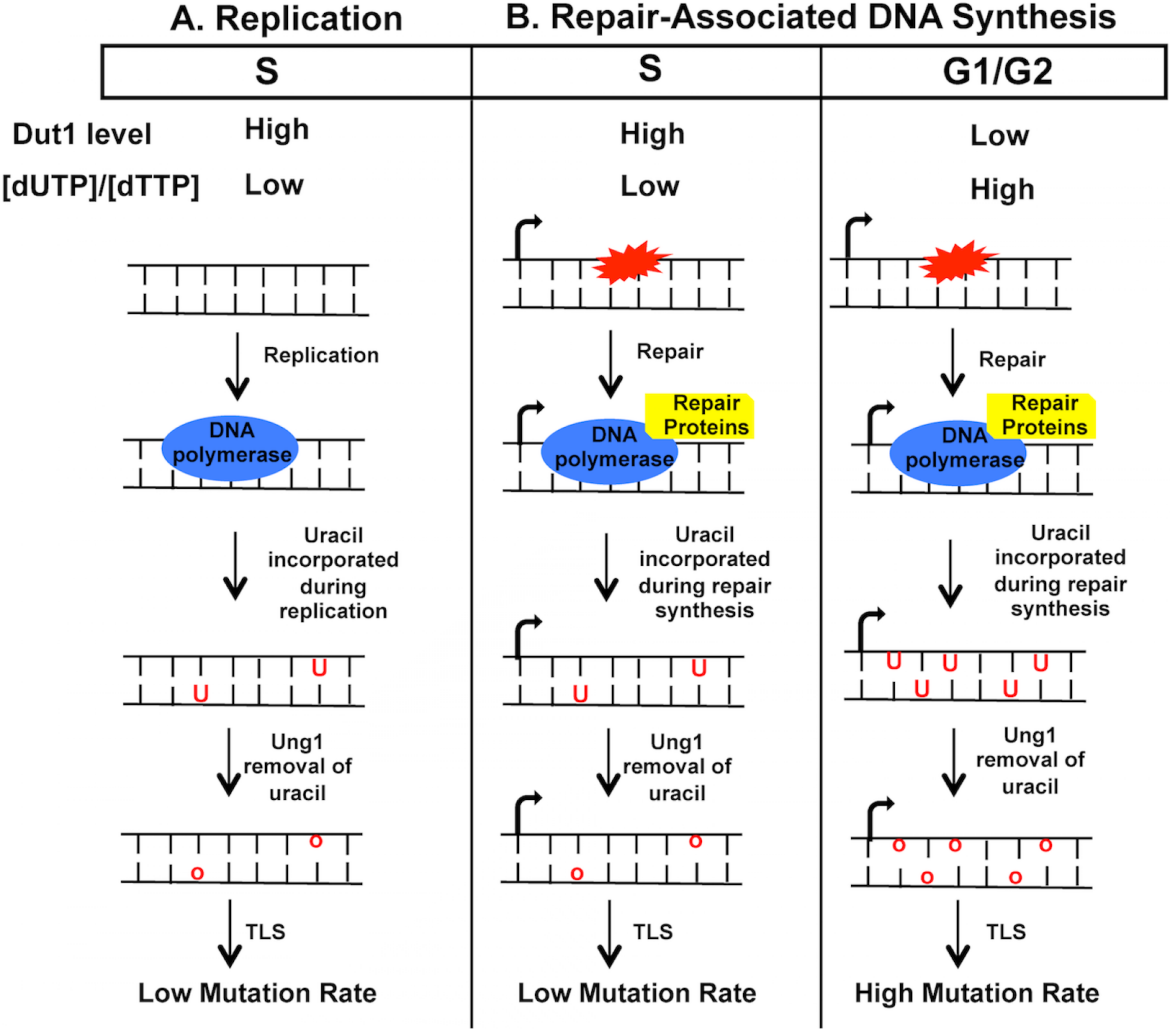
The model of uracil-incorporation into DNA during G1, S, or G2 phases of the cell cycle. Dut1 level is higher and the ratio of [dUTP]/[dTTP] is lower in S phase compared to G1 or G2. **A)** Uracil-incorporation during replication in S phase. The replicative synthesis occurs in S phase; the extent of uracil-incorporation and the ensuing uracil-associated mutagenesis is minor due to the low [dUTP]/[dTTP]. **B)** Uracil-incorporation during the repair-associated DNA synthesis. In S-phase (middle), the repair synthesis induced by transcription-associated endogenous DNA damage is subject to the low [dUTP]/[dTTP]; the extent of uracil-incorporation and the ensuing uracil-associated mutagenesis is minor due to the low [dUTP]/[dTTP]. In G1 or G2 phase, the repair synthesis induced by transcription-associated endogenous DNA damage is subject to the relatively high [dUTP]/[dTTP]; the extent of uracil-incorporation and the ensuing uracil-associated mutagenesis is significant.

The repair-associated DNA synthesis occurring in G1- or G2-phase of cell-cycle would be a particularly potent way of uracil incorporation into DNA because the expression of dUTPase is upregulated in S-phase (S4A Fig and [48–50]). This cell-cycle dependent regulation of the available [dUTP] would ensure the minimal uracil-incorporation into DNA during replication (Fig 8), but comparatively increases the [dUTP]/[dTTP] ratio and thus the possibility of dUTP being used by DNA polymerases during the repair synthesis occurring outside of S-phase. When we lowered the [dUTP]/[dTTP] ratio by overexpressing the dUTPase-encoding gene *DUT1* from the G1- or G2-specific promoters, the rates of mutations at the *pTET-lys2-TAA* reporter under the high transcription conditions in BER-deficient cells were significantly reduced (Fig 7A and 7B), indicating that the uracil incorporated into DNA during G1 or G2 comprise a substantial source of transcription-associated mutations (Fig 8). The *DUT1*-overexpresion from the ubiquitous *pGAL* promoter, however, resulted in much greater reduction in the mutation rate at the *pTET-lys2-TAA* reporter under high transcription conditions. This effect could largely be explained by the higher overall level of expression from *pGAL* promoter but also could indicate that a considerable level of uracil-incorporation does occur in all cell cycles including S. The effect of the *DUT1-*overexpression from S-specific promoter in reducing uracil-associated mutations when the *pTET-lys2-TAA* reporter under high-transcription conditions was relatively smaller compared to the G1- or G2-specific promoters. And the overexpression of *DUT1* from *pGAL* or S-specific promoter as well as G1- or G2-specific promoters had no effect on the mutation rates when the transcription of the *pTET-lys2-TAA* was repressed, indicating that the level of uracil incorporated into DNA during replication, which would be uniform throughout the genome and only affected by the *DUT1*-expression from the *pGAL* or S-specific promoter, cannot induce uracil-associated mutations to a significant degree.

In summary, we found a novel mechanism of introducing uracil into DNA during the damage-induced repair synthesis during G1- or G2-phase of cell cycle. The repair-coupled uracil-incorporation would be a way to non-uniformly alter the nucleotide composition of genomic DNA. The degree of alteration and the extent of uracil-incorporation would depend on the extent of repair synthesis occurrence and would be expected to be greater at regions of frequent endogenous DNA damage i.e. highly transcribed genomic loci. Such a role played by transcription in changing the nucleotide composition locally would apply to other types of non-canonical residues. We speculate that a similar mechanism is involved in specifically elevating the ribonucleotides at highly transcribed regions. Previous studies have shown that ribonucleotide-dependent mutations are highly elevated by transcription [60, 61]. And similar to *DUT1*, *RNR1*, the gene encoding the essential, regulatory subunit of the ribonucleotide reductase, is regulated in a cell-cycle dependent manner to ensure the optimal [dNTP]/[rNTP] ratio for the replication during S-phase [62]. There are several remaining questions to be answered through further studies such as whether the correlation between uracil and transcription apply linearly genome-wide and whether other sources of the endogenous DNA damage such as non-B DNA structures could elevate uracil-content through DNA repair synthesis. Further work is also needed to determine the specific repair pathway directing the uracil incorporation at highly transcribed regions.

## Methods and materials

### Strains and plasmids

Yeast strains used in this study were derived from YPH45 (*MATa*, *ura3-52 ade2-101 trp1Δ1)*. The construction of strains containing the *his4Δ*::*pTET-lys2-TAA* allele was previously described [19]. The *his4Δ*::*pTET-lys2-TAG* allele was introduced by the pop-in/pop-out two-step allele replacement method to replace the *his4Δ*::*pTET-LYS2* allele on Chr III using Bgl*II*-digested pSR982. Further gene deletions of the yeast strains containing the *pTET-lys2*-*TAA* or -*TAG* allele were carried out by the standard one-step gene disruption method. The subsequent Cre/*lox*P-mediated deletion of the marker gene was carried out as appropriate [63].

pCDG is a 2-micron plasmid with a mutant human UDG encoding sequence under the *pGAL* control with the *TRP1* marker [25] and was a gift from Dr. Bruce Demple (Stony Brooke School of Medicine, Stony Brooke, NY). The cell-cycle specific Dut1-overexpression plasmids were constructed by digesting p426-GAL1-DUT1 [18] with BamH*I* and Sac*I* to remove and replace the pGAL promoter with the promoters of yeast genes *CLN2*, *HHO1* or *CLB2*. Sequences of primers used to amplify the promoters of *CLN2*, *HHO1* or *CLB2* genes from the yeast genome were previously described [14, 52]. The 537 nt sequence encoding the *CLN2* PEST domain was synthesized through the Invitrogen GeneArt Gene Synthesis service and was inserted into the EcoR*I*/BamH*I* digested pCLN2-DUT1, pHHO1-DUT1, and pCLB2-DUT1 plasmids. Each of these plasmids were further modified by the addition of 3XHA-encoding sequence to the C-terminal ends.

### Mutation rates, frequency, and spectra

Mutation rates and frequency were determined by fluctuation analysis and the method of the median; the 95% confidence intervals were calculated as previously described [64]. For the CDG expression experiments, the indicated strains were transformed with either the empty vector pYES2 or pCDG and selectively plated on the synthetic complete media with 2% dextrose media lacking tryptophan (SCD-Trp). Individual colonies were inoculated into a 1 mL SC-Trp cultures supplemented with 2% galactose and 1% raffinose. After 4 days of growth at 30°C, appropriate dilutions were plated on SCD-Trp to determine total cell numbers and on SCD-Trp-Lys to determine the number of Lys+ revertants in each culture. For the Dut1-overexpression experiments, the indicated strains were transformed with pRS426 or pGAL-DUT1, pCLN2-DUT1-PEST-HA, pHHO1-DUT1-PEST-HA, or pCLB2-DUT1-PEST-HA. After culturing in SC-Ura media with 2% galactose/1% raffinose or SC-Ura with 2% glycerol/2% ethanol for 4 days at 30°C, the Lys+ revertants were selected on SC-Ura-Lys plates. Where “low transcription” is indicated, 2 μg/mL doxycyline was added to the media.

To determine the mutation frequency following drug treatments, overnight cultures in YEPD (1% yeast extract, 2% peptone, 2% glucose) were diluted to an OD of 0.2 and grown for 4 hrs at 30°C. 5-FU, 4NQO, or CPT was added to the yeast culture to a final concentration of 10 μM, 0.2 μg/mL and 100 μM, respectively, and incubated at 30°C for 20 hours with shaking. Cell pellets were spun and washed twice and plated on SCD-Lys plates and on YEPD plates. Colonies were counted after 48 hrs and the mutation frequency was calculated as described above. The mutation spectra and the 95% confidence intervals for the specific mutation types were determined as previously described [31].

### Quantitative long-amplicon real-time PCR

The uracil density in DNA was quantified using the long amplicon quantitative real-time PCR approach as previously described with the following modifications [65]. 5 μg of each DNA samples were digested with 1 unit of UDG (New England Biolabs) for 30 mins followed by an incubation with EndoVIII (New England Biolabs) for 1 hr at 37°C. After DNA was precipitated and dissolved in water, 100 ng of DNA from each sample was used to carry out qPCR in triplicates. Primers used for the amplification of the yeast *LYS2*, *CAN1*, and *TDH3* are listed in the S7 Table. The amplification was performed using Bioline SensiFAST SYBR No-ROX kit and Biorad CFX Connect Real-Time PCR machine. Cycling parameters were as follows: For the short amplicons: 95°C for 3 min followed by 40 cycles of 95°C for 5 s, 60°C for 10 s and 72°C for 10 s. For the long amplicons: 95°C for 3 min followed by 40 cycles of 95°C for 5 s, 60°C for 10 s and 72°C for 1 min.

### Determination of the induced lesion frequency

The frequency of uracil in DNA was calculated by assuming that the UDG/EndoVIII treatment leads to the strand breaks specifically at the location of uracil, which results in the quantitative loss of the template DNA and consequently the reduced qPCR amplification efficiency. The uracil density at *pTET-LYS2*, *CAN1*, and *TDH3* was inferred from the reduction in the amplification of the UDG/EndoVIII-treated samples relative to the untreated samples when amplifying a large 3 to 4 kb regions at each gene. For each gene, qPCR amplification of ~ 100 bp target area was used to normalize for the template DNA loading. Primers used for the amplification of the 100 bp or the 3 to 4 kb regions of the yeast *LYS2*, *CAN1*, and *TDH3* are listed in the S7 Table. Assuming the Poisson distribution of uracil in the large amplicons, the density of uracil was calculated using the following equation where the amplification percent of the large amplicons in the UDG/EndoVIII-treated and in the untreated controls, relative to the amplification of the small ~100 bp amplicons, are represented by *At* and *Au*, respectively.

$$\mathrm{Uracils}\;\mathrm{per}\;10\mathrm{Kb}\;\mathrm{DNA}=\frac{-\ln(\frac{At}{Au})\;x\;10000bp}{Size\;of\;long\;amplicon\;(bp)}$$

### Labelling and quantification of uracil

Fluorescent labelling of the uracil-derived AP sites was performed as previously described with minor modifications [45]. Briefly, genomic DNA was isolated from the *ung1Δ* yeast cells treated with the indicated concentrations of 5-FU or 4NQO and treated with 10 mM methoxyamine. Then, 5 μg of each DNA sample was treated with 1 unit of UDG (New England Biolabs) for 30 mins at 37°C and labeled by incubation with 5 mM AA3 for an additional hour. Following the addition of Cy5 azide (Lumiprobe) to the final concentration of 0.5 mM followed and the freshly prepared CuBr/TBTA (1:4 in DMSO/t-BuOH 3:1, 0.5 mM, Sigma), the mixture was shaken at 37°C for 2 hrs. The Cy5/AA3-labeled DNA was purified by ethanol precipitation, heated at 95°C, and transferred to a positively charged nylon membrane using the Bio-Dot microfiltration apparatus (Biorad). The membrane was scanned using the ChemiDoc MP imaging system (Biorad) with a Cy5 filter and quantified using the Image Lab software.

### Cell synchronization

Synchronization of yeast cells at G1 was carried out by arresting *bar1Δ* cells with α-factor as previously described [66]. Briefly, yeast cells were grown in either YEPD or SC-Ura + 2% glucose overnight. The overnight cultures were diluted to an OD_600_ of 0.2 (for YPD) or 0.4 (for SC-Ura + glucose) and grown at 30°C until reaching an OD_600_ of 0.8. The cells were washed twice followed by the addition of α-factor (Sigma) to a final concentration of 50ng/mL and grown for 2 hrs or until ~100% of the cells were unbudded with a typical pear/ schmoo shape characteristic of α-factor arrest. To remove the α-factor, cells were washed twice with water and resuspended in the media containing 50 μg/mL pronase. Cells were collected after every 15 minutes (YEPD) or 20 minutes (SC-Ura + 2% glucose) following the release and used for RNA analysis.

### 5-FU and 4-NQO spot and survival assays

The overnight cultures were diluted to an OD_600_ of 0.2 and grown at 30°C for 4 hrs before adding 5-FU, 4NQO or DMSO to a final concentration of 10 μM, 0.2 μg/mL, and 0.1%, respectively. Following incubation at 30°C for 20 hrs with shaking, cells were spun down, washed twice, and plated on YEPD plates. Colonies were counted and the percent survival of 5-FU- or 4NQO-treated cultures were calculated relative to the DMSO-added cultures.

## Supporting information

S1 Table Mutation rates and spectra at the *lys2-TAG* allele in the *apn1Δ* strain. (PDF)

S2 Table Mutation Rates, mutation spectra and fold change following CDG expression. (PDF)

S3 Table Uracil Quantitation using long-amplicon QPCR. (PDF)

S4 Table Mutation frequencies following drug treatment. (PDF)

S5 Table The quantitation of Cy5 Signal following drug treatments. (PDF)

S6 Table Mutation Rates at the *pTET-lys2-TAA* in *apn1 ntg1 ntg2* strains following Dut1 Overexpression. (PDF)

S7 Table Primers used in quantitative real-time PCR analyses. (PDF)

S1 Fig The rate of Lys+ mutations in *apn1Δ dcd1Δ* strain and expression levels of target genes and primer locations used in the long-amplicon qPCR. **A)** Overall mutation rates of the indicated yeast strains under the high transcription conditions (no doxycycline). Error bars indicate 95% confidence intervals. **B)** The expression level of the indicated genes in the presence (+) or absence (-) of doxycycline in *ung1Δ* or *ung1Δ dcd1Δ* strains as determined by qRT-PCR with the *ALG9* gene as the control. Error bars indicate standard deviations and all measurements are from N = 6. **C)** The locations of primer sets used in the long-amplicon qPCR are indicated. The sequences of the primers are listed in S1 Table.
(PDF)

S2 Fig The frequencies A to T mutations following 4NQO treatment. The frequencies of A>T Lys+ mutations following treatments with 0.2 μg/mL 4NQO. Error bars indicate 95% confidence intervals.
(PDF)

S3 Fig Schematic representation of AA3-labeling of AP sites. Uracil is excised by UDG to create AP sites. AP sites are labelled with AA3 followed by reaction with Cy5-azide and quantitation of the fluorescence on a nylon membrane.
(PDF)

S4 Fig *DUT1* expression levels **A)** Relative mRNA level of endogenous *DUT1* or endogenous *HTA2* gene expression in *bar1Δ* cells synchronized with α-factor. RNA was collected every 20 min after the release from α-factor. N = 6 for all data points. **B)** Relative expression level of *DUT1* was measure by qRT-PCR from the asynchronous cells transformed with the plasmids from *pGAL* and the G1-, S-, G2-specific promoters. Error bars indicate standard deviations and all measurements are from N = 3. **C)** Relative mRNA level of endogenous *HTA2* or *DUT1* overexpressed from *pCLN2* promoter, *pHHF01* promoter **(D)** or *pCLB2* promoter **(E).** Expression levels were assessed by qPCR and normalized to *ALG9*. N = 6 for all data points.
(PDF)

S1 File Supplemental materials and methods. (PDF)
