# Supplementary material for: Unscheduled DNA synthesis leads to elevated uracil residues at highly transcribed genomic loci in Saccharomyces cerevisiae
Source: PLoS Genet. 2018 Jul 17;14(7):e1007516. doi: 10.1371/journal.pgen.1007516 (PMC6063437; doi:10.1371/journal.pgen.1007516)
Supplement: S1 Table — (PDF) [file pgen.1007516.s001.pdf]

Table S1: Mutation Rates and Spectra at the *lys2-TAG* allele in the *apn1*Δ strain.

	T	A	G	Mutation Ratex10 $^{-8}$ (95% confidence interval)	N
Mutated sequence	*a*	*A*	*G*	2.92	2
	c	*A*	*G*	2.92	2
	g	*A*	*G*	8.75	6
	T	*c*	*G*	53.9	37
	T	*A*	*c*	1.46	1
**Total**				70 (58.5 – 93.8)	48

Mutated bases are indicated in lower case letters. Uracil-associated and cytosine-associated mutations are highlighted in yellow and blue, respectively.
