# Supplementary material for: Unscheduled DNA synthesis leads to elevated uracil residues at highly transcribed genomic loci in Saccharomyces cerevisiae
Source: PLoS Genet. 2018 Jul 17;14(7):e1007516. doi: 10.1371/journal.pgen.1007516 (PMC6063437; doi:10.1371/journal.pgen.1007516)
Supplement: S2 Table — (PDF) [file pgen.1007516.s002.pdf]

Table S2: Mutation Rates, Mutation Spectra and Fold Change following CDG expression

Relevant Genotype	Mutation Rate (x10 $^{-8}$)	High rate* (95%CI)	N*** (High)	Low rate** (95% CI)	N*** (Low)	Rate ratio (High/Low)
apn1 ung1	**Overall**	18.3 (15-21.8)	91	2.57 (2.11-2.8)	56	7.12
	**A>C**	3.3	25	0.78	17	4.2
	**T>G**	5.14	39	0.46	10	11.2
	**A>C + T>G**	12.9	64	1.24	27	10.4
	**G>C**	0.92	7	0.55	12	1.67
	**Other**	4.02	20	0.78	17	5.15
apn1 ung1 rad14	**Overall**	38.7 (36.8-41.9)	92	4.61 (3.99-6.17)	67	8.4
	**A>C**	18.1	43	1.58	23	11.5
	**T>G**	1.68	4	0.34	5	4.94
	**A>C + T>G**	19.8	47	1.93	28	10.24
	**G>C**	5.05	12	1.99	29	2.53
	**Other**	13.9	33	0.68	10	20.4
apn1 ung1 top1	**Overall**	16.8 (14.5-17.8)	80	2.14 (1.63-2.62)	55	7.85
	**A>C**	4.41	21	0.27	7	16.3
	**T>G**	4.41	21	0.16	4	27.56
	**A>C + T>G**	8.82	42	0.43	11	20.8
	**G>C**	2.94	14	1.17	30	2.52
	**Other**	5.04	24	0.97	25	5.19
apn1 ung1 rad14 top1	**Overall**	40.8 (35-45)	86	5.03 (4.49-5.81)	65	7.95
	**A>C**	18.03	38	0.31	4	58.2
	**T>G**	3.8	8	0.62	8	6.12
	**A>C + T>G**	21.8	46	0.93	12	23.44
	**G>C**	7.11	15	0.93	12	7.64
	**Other**	11.9	25	3.17	41	3.74
apn1 ung1 rnh1 rnh201	**Overall**	12.7 (9.14-14.8)	77	2.01 (1.52-2.41)	75	6.32
	**A>C**	1.81	11	0.40	15	4.5
	**T>G**	3.13	19	0.08	3	39.13
	**A>C + T>G**	4.95	30	0.48	18	10.3
	**G>C**	2.64	16	0.75	28	3.52
	**Other**	5.11	31	0.78	29	6.55

*Rates of mutations under high transcription conditions (no doxycycline)
**Rates of mutations under low transcription conditions (2μg/mL doxycycline)
***Rates of specific mutation types were calculated by multiplying the overall rate by the proportion of the relevant mutation type in the corresponding spectrum.
