# Supplementary material for: Unscheduled DNA synthesis leads to elevated uracil residues at highly transcribed genomic loci in Saccharomyces cerevisiae
Source: PLoS Genet. 2018 Jul 17;14(7):e1007516. doi: 10.1371/journal.pgen.1007516 (PMC6063437; doi:10.1371/journal.pgen.1007516)
Supplement: S3 Table — (PDF) [file pgen.1007516.s003.pdf]

Table S3: Uracil Quantitation Using Long-Amplicon QPCR

	LYS2 3KB		LYS2 4KB	
Conditions	% amplification	Uracil Density	% amplification	Uracil Density
***ung1* + 0uM 5FU**	73.5±6.7	1.01±0.29	73.2±13	1.03±0.42
***ung1* + 1uM 5FU**	78.1±2.8	0.81±0.122	61.7±4.0	1.17±0.16
***ung1* + 5uM 5FU**	51.6±6.1	2.18±0.39	42.3±4.2	2.08±0.26
***ung1* + 10uM 5FU**	49.0±5.1	2.34±0.34	29.3±5.6	3.01±0.45

	**WT**		***ung1***		***ung1 dcd1***	
Primers	-dox	+dox	-dox	+dox	-dox	+dox
**LYS2 3KB**	0.451±0.18	0.072±0.11	1.53±0.18	0.93±0.11	2.05±0.26	1.13±0.18
**LYS2 4KB**	0.526±0.27	0.21±0.22	1.31±0.20	0.64±0.29	2.09±0.34	1.35±0.22
**CAN1 3KB**	0.245±0.21	0.16±0.18	0.26±0.155	0.36±0.37	1.38±0.41	1.34±0.37
**TDH3 3KB**	0.404±0.21	0.51±0.21	0.975±0.35	1.20±0.69	1.6±0.24	1.62±0.30
