# Supplementary material for: Unscheduled DNA synthesis leads to elevated uracil residues at highly transcribed genomic loci in Saccharomyces cerevisiae
Source: PLoS Genet. 2018 Jul 17;14(7):e1007516. doi: 10.1371/journal.pgen.1007516 (PMC6063437; doi:10.1371/journal.pgen.1007516)
Supplement: S4 Table — (PDF) [file pgen.1007516.s004.pdf]

Table S4: Mutation Frequencies Following Drug Treatment.

		DMSO (12 cultures)*		5-FU (12 cultures)*	
		Mutation Frequency x10^{-8} (95% CI)	# of Lys+ sequenced	Mutation Frequency x10^{-8} (95% CI)	# of Lys+ sequenced
WT	Overall	2.17 (1-5.33)	84	2.26 (0.65-5.48)	32
	Uracil**	0.8 (0.32-1.99)	31	1.6 (0.39-3.90)	16
ung1	Overall	4.90 (1.8-7.26)	30	1.98 (1.32-8.25)	34
	Uracil**	0.65 (0.5-1.6)	4	0.48 (0.21-2.04)	10
apn1	Overall	16.9 (6.5-29.4)	86	89.2 (57-102)	72
	Uracil**	15.7 (5.95-27.4)	80	83 (52-95)	67
apn1 ung1	Overall	3.93 (2.5-4.64)	44	3.66 (2.33-4.66)	50
	Uracil**	1.61 (0.8-2.29)	16	2.10 (1.17-2.89)	23
		**DMSO (24 cultures)***		**CPT (24 cultures)***	
		Mutation Frequency x10^{-8} (95% CI)	# of Lys+ sequenced	Mutation Frequency x10^{-8} (95% CI)	# of Lys+ sequenced
WT	Overall	0.51 (0.25-0.75)	84	1.6 (0.97-1.7)	46
	Uracil**	0.19(0.1-0.3)	31	0.73 (0.36-0.97)	21
ung1	Overall	0.57(0.45-0.92)	30	1.91 (1.6-3.19)	54
	Uracil**	0.08 (0.02-0.23)	4	0.6(0.36-1.08)	17
apn1	Overall	2 (1.56-2.89)	86	6.95 (5.83-11.4)	97
	Uracil**	1.86 (1.42-2.69)	80	4.51 (3.47-7.47)	77
apn1 ung1	Overall	0.95 (0.71-1.19)	44	2.76 (1.38-5.52)	98
	Uracil**	0.37 (0.21-0.54)	16	0.87 (0.37-1.79)	31
		**DMSO (24 cultures)***		**4NQO (24 cultures)***	
		Mutation Frequency x10^{-8} (95% CI)	# of Lys+ sequenced	Mutation Frequency x10^{-8} (95% CI)	# of Lys+ sequenced
WT	Overall	1 (0.5-2.0)	84	55.8 (39.9-70)	133
	Uracil**	0.37 (0.15-0.75)	31	7.97 (4.24-12.5)	19
ung1	Overall	0.99 (0.49-1.02)	30	44.8 (37.3-46.4)	123
	Uracil**	0.132 (0.02-0.38)	4	5.46 (2.96-8.78)	14
apn1	Overall	7.93 (2.64-11.6)	86	122 (83-166)	168
	Uracil**	7.38 (2.4-10.8)	80	29.8 (17-44)	41
apn1 ung1	Overall	1.16 (1.74-0.58)	44	59.4 (33-81.4)	146
	Uracil**	0.47 (0.187-0.78)	16	7.32 (3.02-12.1)	18

*Numbers of the cultures used to determine the mutation frequencies.
**The frequencies of uracil-dependent mutations were determined by multiplying the proportion of the A>C and T>G mutations with the overall mutation frequencies.
