# Supplementary material for: Unscheduled DNA synthesis leads to elevated uracil residues at highly transcribed genomic loci in Saccharomyces cerevisiae
Source: PLoS Genet. 2018 Jul 17;14(7):e1007516. doi: 10.1371/journal.pgen.1007516 (PMC6063437; doi:10.1371/journal.pgen.1007516)
Supplement: S5 Table — (PDF) [file pgen.1007516.s005.pdf]

Table S5: The quantitation of Cy5 Signal following Drug Treatments

	**Relative Cy5 signal**<br>**N=6 (**± standard deviation)
ung1 + DMSO	1 ± 0.000
ung1 + 10uM 5-FU	1.26 ± 0.145
ung1 + 50uM 5-FU	1.73 ± 0.07
ung1 + 100uM 5-FU	3.42 ± 0.130
Hela cells	0.63 ± 0.030
Daudi cells	3.68 ± 0.120
ung1 + DMSO	1 ± 0.000
ung1 + 1ug/mL 4NQO	1.1 ± 0.095
ung1 + 5ug/mL 4NQO	1.15 ± 0.235
ung1 + 10ug/mL 4NQO	2.26 ± 0.325
ung1 + 20ug/mL 4NQO	3.25 ± 0.495
