# Supplementary material for: Unscheduled DNA synthesis leads to elevated uracil residues at highly transcribed genomic loci in Saccharomyces cerevisiae
Source: PLoS Genet. 2018 Jul 17;14(7):e1007516. doi: 10.1371/journal.pgen.1007516 (PMC6063437; doi:10.1371/journal.pgen.1007516)
Supplement: S6 Table — (PDF) [file pgen.1007516.s006.pdf]

**Table S6: Mutation Rates at the *pTET-lys2-TAA* in *apn1 ntg1 ntg2* strains following Dut1 Overexpression**

| **Galactose + Raffinose (N= 24 cultures)** | | |
|---|---|---|
| | Mutation Rate ( x$10^{-8}$) (High Transcription) | Mutation Rate ( x$10^{-8}$) Low Transcription |
| Vector | 180 (149-212) | 2.24 (1.25-2.54) |
| pGal-DUT1 | 18.3 (12.5-23.5) | 2.26 (1.51-3.93) |
| G1-DUT1 | 94 (77-124) | 2.36 (1.10-3.44) |
| S-DUT1 | 124 (100-171) | 2.27 (1.11-2.53) |
| G2-DUT1 | 115 (96.2-136) | 1.90 (1.58-2.5) |
| | | |
| **Glycerol + Ethanol (N=24 cultures)** | | |
| | Mutation Rate ( x$10^{-8}$) (High Transcription) | Mutation Rate ( x$10^{-8}$) Low Transcription |
| Vector | 70.9 (67.2-74) | 2.94 (2.34-4.40) |
| G1-DUT1 | 45.2 (37.9 – 50.4) | 2.14 (1.46-3.72) |
| S-DUT1 | 54.6 (47-61.7) | 2.96 (2.21-3.26) |
| G2-DUT1 | 50 (46.4-60.3) | 2.13 (1.21-5.43) |
