# Supplementary material for: Unscheduled DNA synthesis leads to elevated uracil residues at highly transcribed genomic loci in Saccharomyces cerevisiae
Source: PLoS Genet. 2018 Jul 17;14(7):e1007516. doi: 10.1371/journal.pgen.1007516 (PMC6063437; doi:10.1371/journal.pgen.1007516)
Supplement: S7 Table — (PDF) [file pgen.1007516.s007.pdf]

Table S7: Primers used in quantitative real-time PCR analyses.

Primer name	Forward primer	Reverse primer	Amplicon Size (nt)
	**For Long-Amplicon qPCR (5' – 3')**		
LYS2 100bp	GAGTAACCGGTGACGATGATATT	CATTAAATGACCACGTTGGTTGA	97
LYS2 3kb	CTTTCAGTGTTACCACATGA	CAAATTTTTCGTTCCAAGTACC	3031
LYS2 4kb	CTTTCAGTGTTACCACATGA	GTTCTATACTTGGCAGTGGAAG	4045
CAN1 100bp	GAGTTCTGGGTCGCTTCCAT	GGCACCTGGGTTTCTCCAAT	120
CAN1 3kb	CAGTCCTATTCGGAGATACAG	CTAACTCAGACATTATCGGAAC	3067
TDH3 100bp	CATGGGGTTCTTCCAACGTTG	GGAAGATGGAGCAGTGATAAC	122
TDH3 3kb	GTTCTCACAC GGAACACCAC-	GTGGCAGCAAGTGATAAGCAAGC	3127
	**For qRT-PCR (5' – 3')**		
DUT1	GGTTCTGCCACTGCCGCGGG	GGCGCAATACGACCGTAGGT	
ALG9	CACGGATAGTGGCTTTGGTGAACAATTAC	TATGATTATCTGGCAGCAGGAAAGAACTTGGG	
HTA2	CCCAGTTGGTAGAGTGCACAG	CTCTAGCAGCATTACCAGCC	
