# Supplementary figures and images for: Unscheduled DNA synthesis leads to elevated uracil residues at highly transcribed genomic loci in Saccharomyces cerevisiae
Source: PLoS Genet. 2018 Jul 17;14(7):e1007516. doi: 10.1371/journal.pgen.1007516 (PMC6063437; doi:10.1371/journal.pgen.1007516)

**Fig. S1**

**A. Overall mutation rate**

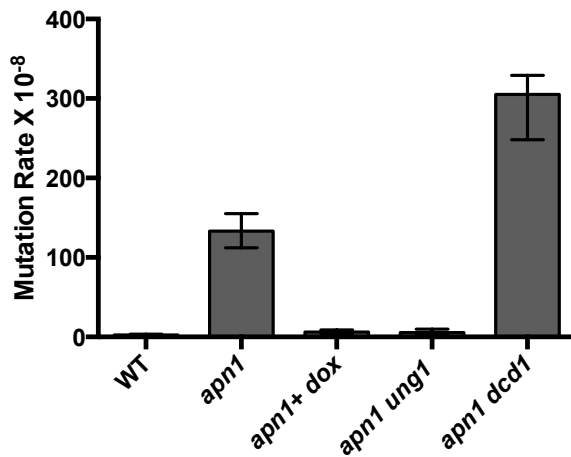

**B. mRNA expression levels +/- doxycycline**

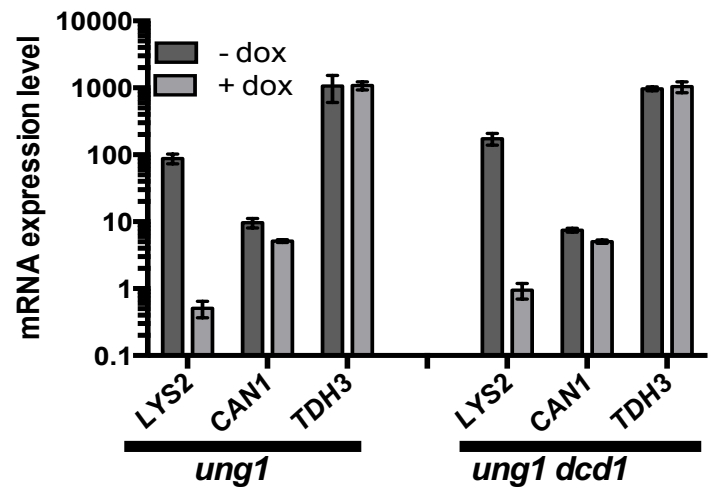

**C. Primer sets for long-QPCR**

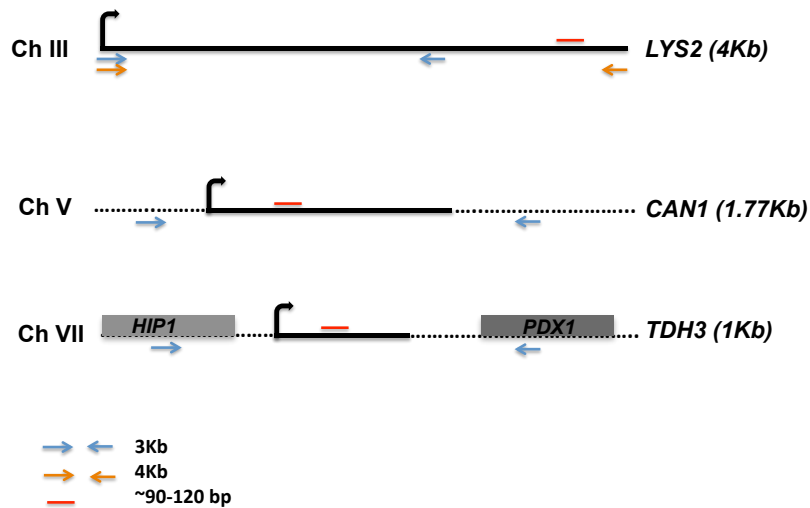

Supplement: S1 Fig — A) Overall mutation rates of the indicated yeast strains under the high transcription conditions (no doxycycline). Error bars indicate 95% confidence intervals. B) The expression level of the indicated genes in the presence (+) or absence (-) of doxycycline in ung1Δ or ung1Δ dcd1Δ strains as determined by qRT-PCR with the ALG9 gene as the control. Error bars indicate standard deviations and all measurements are from N = 6. C) The locations of primer sets used in the long-amplicon qPCR are indicated. The sequences of the primers are listed in S1 Table. (PDF) [file pgen.1007516.s008.pdf]

Fig. S2

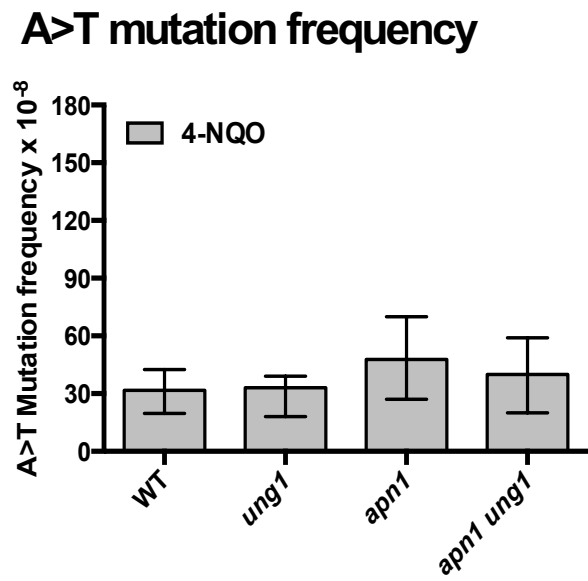

Supplement: S2 Fig — The frequencies of A>T Lys+ mutations following treatments with 0.2 μg/mL 4NQO. Error bars indicate 95% confidence intervals. (PDF) [file pgen.1007516.s009.pdf]
