# Supplementary material for: Unscheduled DNA synthesis leads to elevated uracil residues at highly transcribed genomic loci in Saccharomyces cerevisiae
Source: PLoS Genet. 2018 Jul 17;14(7):e1007516. doi: 10.1371/journal.pgen.1007516 (PMC6063437; doi:10.1371/journal.pgen.1007516)

Fig. S3

Schematic for AA3 labeling

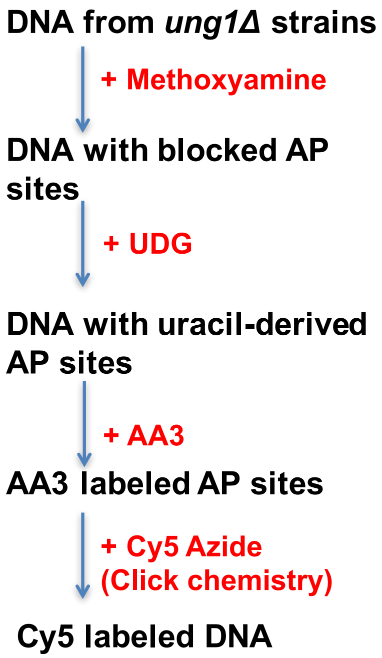

Supplement: S3 Fig — Uracil is excised by UDG to create AP sites. AP sites are labelled with AA3 followed by reaction with Cy5-azide and quantitation of the fluorescence on a nylon membrane. (PDF) [file pgen.1007516.s010.pdf]
