# Supplementary material for: Unscheduled DNA synthesis leads to elevated uracil residues at highly transcribed genomic loci in Saccharomyces cerevisiae
Source: PLoS Genet. 2018 Jul 17;14(7):e1007516. doi: 10.1371/journal.pgen.1007516 (PMC6063437; doi:10.1371/journal.pgen.1007516)

Fig. S4

A. Genomic *DUT1* mRNA level

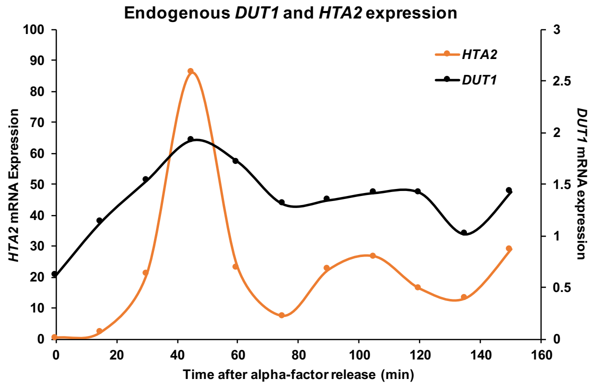

B. Relative DUT1 mRNA Expression (Asynchronous)

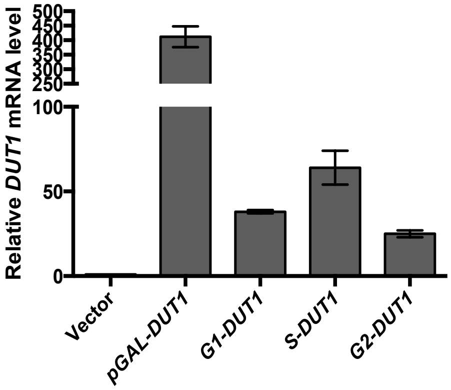

C. G1-*DUT1* mRNA level

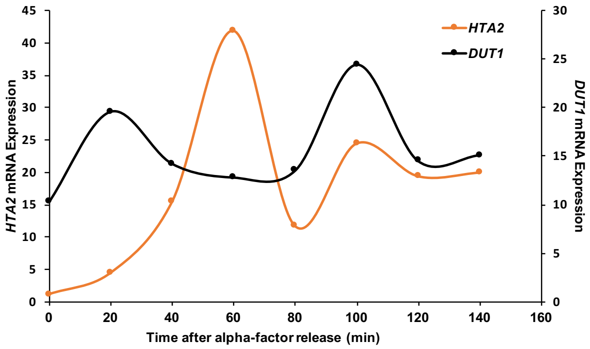

D. S-*DUT1* mRNA level

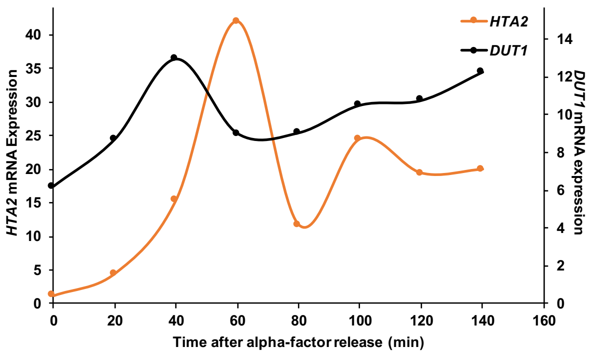

E. G2-*DUT1* mRNA level

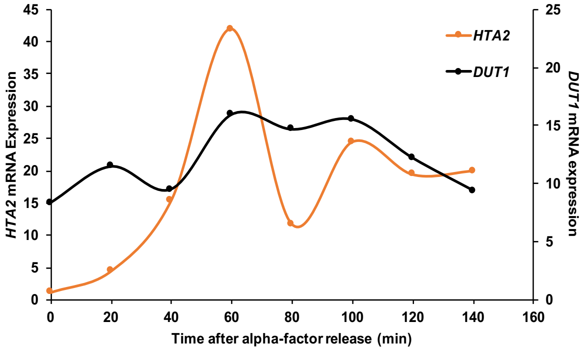

Supplement: S4 Fig — DUT1 expression levels A) Relative mRNA level of endogenous DUT1 or endogenous HTA2 gene expression in bar1Δ cells synchronized with α-factor. RNA was collected every 20 min after the release from α-factor. N = 6 for all data points. B) Relative expression level of DUT1 was measure by qRT-PCR from the asynchronous cells transformed with the plasmids from pGAL and the G1-, S-, G2-specific promoters. Error bars indicate standard deviations and all measurements are from N = 3. C) Relative mRNA level of endogenous HTA2 or DUT1 overexpressed from pCLN2 promoter, pHHF01 promoter (D) or pCLB2 promoter (E). Expression levels were assessed by qPCR and normalized to ALG9. N = 6 for all data points. (PDF) [file pgen.1007516.s011.pdf]
