# Supplementary material for: Unscheduled DNA synthesis leads to elevated uracil residues at highly transcribed genomic loci in Saccharomyces cerevisiae
Source: PLoS Genet. 2018 Jul 17;14(7):e1007516. doi: 10.1371/journal.pgen.1007516 (PMC6063437; doi:10.1371/journal.pgen.1007516)
Supplement: S1 File — (PDF) [file pgen.1007516.s012.pdf]

Supplemental materials and methods

qRT-PCR: Total RNA was extracted using the standard hot acid phenol method and treated with DNase 1 (New England Biolabs). cDNA synthesized using the AmfiRivert cDNA Synthesis Platinum Master Mix from GenDepot was used in the subsequent qPCR performed using amfiSure qGreen Master Mix from GenDepot and Biorad CFX Connect instrument. The qRT-PCR conditions were as follows: 45 °C for 10 mins and 95 °C for 2 mins followed by 40 cycles of 95 °C for 5 s, 60 °C for 10 s and 72 °C for 5 s. The primers used for amplification are listed in Table S7. Relative RNA levels were determined by *ΔΔCq* analysis using *ALG9* as the reference gene.
